# Effect of Carnosine or *β*-Alanine Supplementation on Markers of Glycemic Control and Insulin Resistance in Humans and Animals: A Systematic Review and Meta-analysis

**DOI:** 10.1093/advances/nmab087

**Published:** 2021-07-31

**Authors:** Joseph J Matthews, Eimear Dolan, Paul A Swinton, Lívia Santos, Guilherme G Artioli, Mark D Turner, Kirsty J Elliott-Sale, Craig Sale

**Affiliations:** Sport, Health, and Performance Enhancement (SHAPE) Research Centre, Musculoskeletal Physiology Research Group, School of Science and Technology, Nottingham Trent University, Nottingham, United Kingdom; Research Centre for Life and Sport Sciences (CLaSS), School of Health and Life Sciences, Department of Sport and Exercise, Birmingham City University, Birmingham, United Kingdom; Applied Physiology and Nutrition Research Group, School of Physical Education and Sport, University of Sao Paulo, Sao Paulo, Brazil; School of Health Sciences, Robert Gordon University, Aberdeen, United Kingdom; Sport, Health, and Performance Enhancement (SHAPE) Research Centre, Musculoskeletal Physiology Research Group, School of Science and Technology, Nottingham Trent University, Nottingham, United Kingdom; Applied Physiology and Nutrition Research Group, School of Physical Education and Sport, University of Sao Paulo, Sao Paulo, Brazil; Rheumatology Division, Faculdade de Medicina FMUSP, Universidade de São Paulo, São Paulo, Brazil; Centre for Diabetes, Chronic Diseases, and Ageing, School of Science and Technology, Nottingham Trent University, Nottingham, United Kingdom; Sport, Health, and Performance Enhancement (SHAPE) Research Centre, Musculoskeletal Physiology Research Group, School of Science and Technology, Nottingham Trent University, Nottingham, United Kingdom; Sport, Health, and Performance Enhancement (SHAPE) Research Centre, Musculoskeletal Physiology Research Group, School of Science and Technology, Nottingham Trent University, Nottingham, United Kingdom

**Keywords:** endocrinology, histidine, metabolic health, metabolism, nutrition, obesity

## Abstract

There is growing evidence that supplementation with carnosine, or its rate-limiting precursor *β*-alanine, can ameliorate aspects of metabolic dysregulation that occur in diabetes and its related conditions. The purpose of this systematic review and meta-analysis was to evaluate the effect of carnosine or *β*-alanine supplementation on markers of glycemic control and insulin resistance in humans and animals. We performed a systematic search of 6 electronic databases up to 31 December 2020. Primary outcomes were changes in *1*) fasting glucose, *2*) glycated hemoglobin (HbA1c), and *3*) 2-h glucose following a glucose-tolerance test. A set of additional outcomes included fasting insulin and homeostatic model assessment of β-cell function (HOMA-*β*) and insulin resistance (HOMA-IR). We assessed risk of bias using the Cochrane risk of bias (RoB) 2.0 (human studies) and the Systematic Review Center for Laboratory Animal Experimentation (SYRCLE) RoB (animal studies) tools; and used the Grading of Recommendations Assessment, Development, and Evaluation (GRADE) approach to assess certainty. We used Bayesian hierarchical random-effects models, with informative priors for human data and noninformative priors for animal data. Inferences were made on posterior samples generated by Hamiltonian Markov Chain Monte Carlo using 90% credible intervals (90% CrI) and calculated probabilities. Twenty studies (*n* = 4 human, *n* = 16 rodent) were included, providing data for 2 primary outcomes (fasting glucose and HbA1c) and 3 additional outcomes (fasting insulin, HOMA-*β*, and HOMA-IR). The model provides evidence that supplementation decreases fasting glucose [humans: mean difference (MD)_0.5_ = –0.95 mmol · L^–1^ (90% CrI: –2.1, 0.08); rodent: MD_0.5_ = –2.26 mmol · L^–1^ (90% CrI: –4.03, –0.44)], HbA1c [humans: MD_0.5_ = –0.91% (90% CrI: –1.46, –0.39); rodents: MD_0.5_ = –1.05% (90% CrI: –1.64, –0.52)], HOMA-IR [humans: standardized mean difference (SMD)_0.5_ = –0.41 (90% CrI: –0.82, –0.07); rodents: SMD_0.5_ = –0.63 (90% CrI: –1.98, 0.65)], and fasting insulin [humans: SMD_0.5_ = –0.41 (90% CrI: –0.77, –0.07)]. GRADE assessment showed our certainty in the effect estimate of each outcome to be moderate (human outcomes) or very low (rodent outcomes). Supplementation with carnosine or *β*-alanine may reduce fasting glucose, HbA1c, and HOMA-IR in humans and rodents, and fasting insulin in humans; both compounds show potential as therapeutics to improve glycemic control and insulin resistance. This review was registered at PROSPERO as CRD42020191588.

## Teaser

**Statement of Significance**: This study includes all available human and animal data to provide the most comprehensive assessment to date of the effects of carnosine and *β*-alanine supplementation on glycemic control and insulin resistance.

## Introduction

Diabetes is a major public health problem; worldwide estimates show that 463 million people were living with diabetes in 2019—equivalent to 9.3% of the global population (1). Type 2 diabetes accounts for >90% of these cases, with the remaining made up of type 1 diabetes, gestational diabetes, and rarer types of diabetes (e.g., maturity-onset diabetes of the young). A hallmark of type 2 diabetes is poor glycemic control and insulin resistance (2), which present earlier in life as impaired fasting glucose or impaired glucose tolerance (also known as prediabetes). This represents a high-risk state that requires intervention, as a 45-y-old with prediabetes has a 74% lifetime risk of progression to type 2 diabetes (3). While lifestyle modifications are central to risk reduction, they can be challenging to implement, and long-term adherence limits their effectiveness (4). It is therefore essential to develop low-cost, novel therapies to improve glycemic control and help prevent or delay disease progression.

The multifunctional dipeptide carnosine is an emerging therapeutic that has the potential to contribute to the treatment or management of various chronic diseases (5). Carnosine is a member of the histidine-containing dipeptide (HCD) family and exists naturally in high concentrations in skeletal muscle, with smaller amounts in other excitable tissues (6–9). Dietary sources include meat, poultry, fish, and prawns (10); but the most efficient way to increase tissue stores is by supplementing with carnosine or its rate-limiting precursor *β*-alanine (11). Work from our research group shows that treatment with carnosine recovers glucolipotoxic inhibition of insulin-stimulated glucose uptake in skeletal muscle cells and decreases highly toxic lipid peroxidation products in pancreatic *β*-cells, leading to an increase in insulin secretion (12). Further evidence supports the role of carnosine in nonenzymatic detoxification of reactive aldehydes (13, 14), an effect that *β*-alanine supplementation potentiates in humans (15, 16). This has important clinical implications, as reactive aldehydes have been implicated in the etiology of diabetes (17). Collectively, this suggests that carnosine may be able to ameliorate aspects of the metabolic dysregulation that occurs in diabetes and its related conditions.

There is growing evidence from rodent studies that carnosine supplementation can prevent or delay the development of type 2 diabetes (18, 19). Initial human trials also show promise (20, 21), but there is currently no consensus on whether carnosine or *β*-alanine can be used to treat or manage diabetes. A recent meta-analysis of human studies sought to address this knowledge gap and concluded that supplementation with HCDs improved waist circumference, fasting glucose, and glycated hemoglobin (HbA1c) (22). The review, however, had several methodological shortcomings [for a commentary, see (23)], which included combining effects from studies using multi-ingredient supplements with those supplementing carnosine or *β*-alanine alone. They also included studies supplementing histidine in isolation, which is not a member of the HCD family, and is not rate limiting for carnosine synthesis in humans—at least under standard dietary conditions (24–27). This approach cannot explain whether the beneficial effects are due to carnosine, *β*-alanine, HCDs, or another supplement ingredient. Another meta-analysis on the topic also had methodological issues (28), which included an incomplete and inconsistent risk of bias assessment and the inclusion of the same study sample as 2 separate studies. It is also important to consider outcomes from animal studies, which can provide mechanistic insight and inform future human trials. Therefore, the purpose of this systematic review and meta-analysis was to evaluate the effect of carnosine or *β*-alanine supplementation on markers of glycemic control and insulin resistance in humans and animals.

## Methods

The methods for this study were published in full as a protocol paper (29) and preregistered on PROSPERO (CRD42020191588). Our reporting follows the updated 2020 Preferred Reporting Items for Systematic review and Meta-Analysis (PRISMA) guidelines (30).

### Eligibility criteria

**Table 1** outlines the eligibility criteria. The primary outcomes were changes in fasting glucose (includes plasma, serum, and blood glucose values), HbA1c, and 2-h glucose following a glucose-tolerance test (GTT). These outcomes represent the 3 clinical markers used in the diagnosis of type 1 diabetes, type 2 diabetes, prediabetes, and gestational diabetes (31, 32). A set of additional outcomes included changes in other markers of glycemic control and insulin resistance (Table 1). There were no restrictions on the timing or duration of supplementation, or on the study setting. We included English and non–English-language sources with the latter translated into English using freely available online tools (i.e., Google Translate).

**TABLE 1:** Overview of PICOS eligibility criteria^1^

	Criteria
Participants	Humans with type 1 diabetes, type 2 diabetes, prediabetes, gestational diabetes, impaired fasting glucose, or impaired glucose tolerance [according to WHO guidelines (31, 32)], or with overweight/obesity (BMI ≥ 25 kg/m^2^) where the relevant outcomes were collected and reported
	Animal studies using a diabetes-related disease model (see human criteria), or overweight/obese animals where the relevant outcomes were reported
	No restrictions were applied on age or comorbidities, or on the methods used to induce disease in animal studies
Intervention	Supplementation with carnosine or *β*-alanine. We excluded studies that used a multi-ingredient supplement intervention
	Human studies included oral administration only, whereas animal studies also included administration by other means (e.g., intraperitoneal or intravenous injection)
Comparator	Comparisons for human studies were between placebo and the experimental intervention
	Comparisons for animal studies were between placebo or control (no intervention) and the experimental intervention
	We excluded studies without a control or placebo group
Outcomes	Outcomes relating to glycemic control and insulin resistance: fasting glucose, HbA1c, 2-h glucose following a GTT, fasting insulin, C-peptide, homeostatic model assessment (HOMA) parameters (e.g., HOMA-IR, HOMA-*β*, HOMA-*S*)
Study designs	Studies were limited to nonrandomized and RCTs, including cluster RCTs. We excluded cohort studies, cross-sectional studies, case series, case reports, commentary, and review articles

1 GGT, glucose-tolerance test; HbA1c, glycated hemoglobin; HOMA-*β*, homeostatic model assessment of *β*-cell function; HOMA-IR, homeostatic model assessment of insulin resistance; HOMA-*S*, homeostatic model assessment of insulin sensitivity; PICOS, Participant, Intervention, Comparator, Outcomes, Study designs; RCT, randomized controlled trial.

### Information sources

We searched 6 electronic databases for potentially eligible studies—PubMed, Scopus, Web of Science, Cochrane Central Register of Controlled Trials (CENTRAL), Cumulative Index to Nursing and Allied Health Literature (CINAHL), and ProQuest—from the earliest record in each database up to 31 December 2020. This was supplemented by searching for trial protocols, reference lists and citation tracking of included studies, and relevant reviews. The authors also searched their personal files to identify any additional relevant material.

### Search strategy and selection process

Search strategies were developed using key text words and medical subject headings (MeSH) related to the population, intervention, and outcomes. An academic librarian, not otherwise associated with the project, reviewed all searches using the Peer Review of Electronic Search Strategies (PRESS) checklist (33). The full search strategy for each database and the completed PRESS report are available in the **Supplemental Methods**. Two reviewers independently completed the initial searches (JJM and KJE-S), data extraction (JJM and ED), and assessment of risk of bias (JJM and ED); and 3 reviewers independently completed full-text screening (JJM, LS, and GGA). Disagreements for searches, data extraction, and risk of bias were referred to a third reviewer (CS) who provided a recommendation.

Titles and abstracts of articles from the initial searches were imported into a systematic review management platform (Covidence; Veritas Health Innovation Ltd.); duplicates were removed and remaining articles screened for potential eligibility. We obtained full texts for all articles that appeared to meet the inclusion criteria or where there was any uncertainty; multiple reports of the same study were handled by including the article that provided the most relevant outcome data. Reviewers used the reference manager functions to highlight eligibility criteria and add comments on each article to cross-reference decisions in the event of a disagreement. We contacted study authors to resolve issues regarding eligibility—for example, to clarify methods or obtain necessary data (maximum of 3 e-mail attempts). Reviewers were not blinded to journal titles or the study authors.

### Data-collection process and items

We extracted data using a standardized spreadsheet based upon the Cochrane data collection form for intervention reviews (34). Data items included the following: *1*) study characteristics (location, setting, study design, size, duration, funding sources, and study aim), *2*) human participant characteristics (age, height, sex, body mass, BMI, body fat %, type and duration of condition, activity and exercise levels, and dietary information), *3*) animal characteristics (age, body mass, source, species, strain, sex, genetic modification status, type and duration of condition, method used to induce disease, and housing conditions), *4*) intervention characteristics (name, type of control used, dosage, frequency, duration, route of administration), *5*) outcome characteristics (type of measure; sample sizes; baseline, interim, and postintervention measures of central tendency and dispersion; adherence to the intervention; dropouts; number and nature of side effects; and assessment of blinding to the intervention), and *6*) information relevant to risk of bias and certainty assessment. We converted glucose values to millimoles per liter using a standard equation [mmol · L^–1^ = mg · dL^–1^ × 0.0555; (35)]. We converted all supplement doses to relative cumulative intake [mg · kg body weight (bw)^–1^]. Some animal studies reported the treatment dose as grams per liter dissolved in drinking water, which we multiplied by reported or normative drinking volumes, before converting to an estimate of relative cumulate intake. Where necessary, we extracted measures of central tendency and dispersion from figures using WebPlotDigitizer version 3.10 (https://apps.automeris.io/wpd/) or contacted study authors for additional data (maximum of 3 e-mail attempts). We converted SE to SD using a standard equation (SD = SE × √*n*).

### Risk of bias assessment

#### Study risk of bias

We assessed risk of bias in human studies using the Cochrane risk of bias 2.0 tool (RoB 2) per protocol for parallel-group randomized trials (36), and in animal studies using the Systematic Review Centre for Laboratory Animal Experimentation (SYRCLE) tool (37). Reviewers assessed each study item as either “high risk,” “low risk,” “some concerns” (RoB 2), or “unclear risk” (SYRCLE) of bias. For human studies, we performed a summary judgment for overall risk of bias based upon RoB 2 recommendations.

#### Reporting bias

For human studies, we screened clinical trial registers to compare outcomes reported in the protocol with each published report. For animal studies, or where there was no preregistration or protocol, we compared the outcomes reported in the methods with the results section of each study. Small study bias, including publication bias, was explored by visually inspecting funnel plots and, where substantive asymmetry was present, conducting a multilevel extension of Egger's regression test (38).

### Effect measures

We extracted and analyzed all outcomes as continuous measures. Mean difference (MD) effect sizes (not standardized) were calculated for the primary outcomes: modeling outcomes on the same absolute scale as the original measurement provides more clinically interpretable results. We also identified minimal important difference thresholds (fasting glucose: 1 mmol · L^–1^ reduction; HbA1c: 0.5% reduction) (39) and calculated the probability that the pooled effect size met or exceeded threshold values (see Data synthesis section). For additional outcomes, MD effect size estimates were standardized (SMD) using reported SDs to account for differences in measurement scales. We used standard threshold values of 0.2, 0.5, and 0.8 to describe effect size estimates as small, medium, and large (40), with values between 0 and 0.2 described as trivial. Data collected from human studies included both baseline and postintervention values and effect sizes were calculated with both sets of information (41), whereas effect sizes were calculated from postintervention values only in animal studies (42).

### Data synthesis

Meta-analyses were conducted within a Bayesian framework, providing a flexible modeling approach to account for uncertainty in model parameters and underlying structures within the data. Bayesian models enable intuitive interpretation of results through reporting subjective probabilities rather than null hypothesis tests or frequentist confidence intervals (43). We planned to conduct 3-level Bayesian hierarchical models with noninformative priors for the between-study variance parameters and adhered to this for analyses of animal data. Due to limitations in the number of human studies and effect sizes, a deviation from the original protocol (29) was required. Instead, standard (2-level) Bayesian random-effects models were conducted, and an informative log-t distribution used as a prior for the between-study heterogeneity variance using a predictive distribution provided for biological markers in pharmacologic versus placebo or control studies (44). Informative priors were used in the human data to estimate the within-study variances and account for unknown correlations between baseline and postintervention values. This was achieved by assuming a uniform prior for each within-study variance based upon a correlation ranging from 0.5 to 0.9. Inferences from all analyses were performed on posterior samples generated by Hamiltonian Markov chain Monte Carlo simulations, and through use of the median value (0.5-quantile) and 90% credible intervals (90% CrI) and calculated probabilities.

Sensitivity and subgroup analyses were performed to examine the robustness of the main model results. Preplanned analyses included removing studies at high risk of bias, as well as meta-regressions to explore the effect of type of supplementation (carnosine or *β*-alanine), duration of supplementation, and the disease type. Following data extraction, additional sensitivity analyses were performed for clear outliers and for studies where the outcomes of interest were not elevated at baseline (human studies) or in the control group (animal studies). Due to heterogeneity across animal studies, meta-regressions for the dose-response of relative cumulative intake were also performed. Analyses were performed using R2OpenBUGS (45) and the R wrapper package brms interfaced with Stan to perform sampling (46).

### Certainty assessment

The certainty of each outcome was assessed using the Grading of Recommendations Assessment, Development, and Evaluation (GRADE) approach (47), across 5 domains: risk of bias, inconsistency, indirectness, imprecision, and publication bias. Outcomes from human trials began with a high-quality rating, based upon their randomized controlled trial (RCT) design (as indicated by the eligibility criteria). This initial rating was subsequently maintained, or downgraded, based upon performance in each of the 5 domains, resulting in an overall rating of high, moderate, low, or very low for each outcome (47). Inconsistency was graded on visual inspection of effect size estimates, whether credible intervals overlapped, and between-study variability [τ (tau)]; these factors were considered within the context of the outcome values at baseline and the cumulative supplement dose, which could plausibly explain inconsistency (48). Human studies were not downgraded for indirectness, as our eligibility criteria narrowly selected for the population, intervention, and outcomes of interest; further, our primary outcomes are indirect by nature and used in clinical decision making. Animal studies were automatically graded down 1 level for indirectness, unless conducted in nonhuman primates (49). Imprecision was graded based on the pooled sample size and the width of the credible intervals: the interval crossed the null and simultaneously included large clinical benefit or harm (serious), or the interval included both a large clinical benefit and harm (very serious) (48, 49). Publication bias was graded as either detected or undetected (see “Outcome reporting bias”).

## Results

### Study selection and characteristics

**FIGURE 1:**
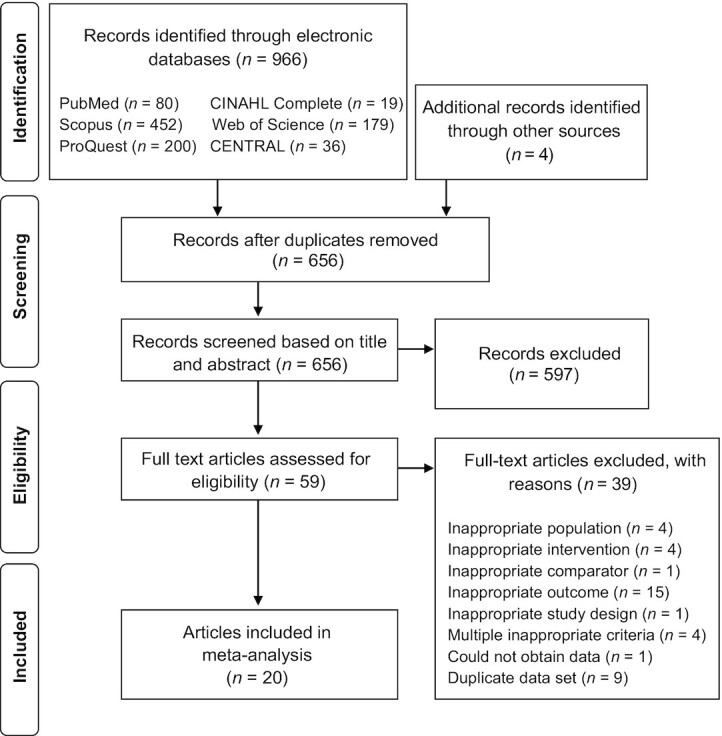
PRISMA flow diagram depicting the search and selection process. PRISMA, Preferred Reporting Items for Systematic review and Meta-Analysis.

**Figure 1** depicts the search and selection process. Twenty studies were included in the data synthesis—7 studies in mice (*n* = 132 mice), 9 studies in rats (*n* = 159 rats), and 4 human studies (*n* = 172 participants)—providing data for 2 primary outcomes (fasting glucose and HbA1c) and 3 additional outcomes [fasting insulin, HOMA-IR, and homeostatic model assessment for steady-state *β*-cell function (HOMA-*β*)]. As all included animal studies were conducted in mice or rats, the term rodent(s) is used herein instead of the nonspecific term animal(s). **Tables 2** and **3** summarize the characteristics of included human and rodent studies. Human populations included adults with type 2 diabetes (21, 50), children with type 1 diabetes (51), and nondiabetic adults with overweight or obesity (a subgroup exhibited impaired glucose tolerance) (20). Rodent disease models included *1*) genetic modifications to develop obesity, hyperglycemia, and insulin resistance (18, 19, 52, 53); *2*) dietary interventions to develop obesity, hyperglycemia or hyperinsulinemia, and insulin resistance (54–56); and *3*) single or multiple streptozotocin injection(s) to induce pancreatic *β*-cell death, leading to hyperglycemia (57–65).

**TABLE 2:** Characteristics and outcomes for included human studies^1^

Study (reference), country	Design and setting	Population	Participant characteristics (F/M, *n*/*n*)	Intervention	Extracted outcomes
de Courten et al. (20), Slovakia	RCT, research institute	*n* = 30 Nondiabetic, sedentary individuals with overweight or obesity	Int: *n* = 15 (3/12)	Int: carnosineCon: placebo (sucrose) 2 g · d^−1^ for 12 wk (2 × 1-g doses)	FG (mmol · L^−1^)
			Age: 42 ± 7 y		FI (mU · L^−1^)
			BMI (kg/m^2^): 31.1 ± 4.6		HbA1c (%)
			Con: *n* = 11 (4/7)		HOMA-*β* (%)
			Age: 43 ± 10 y		HOMA-IR
			BMI: 31.6 ± 3.7		
Elbarbary et al. (51), Egypt	RCT, hospital: pediatric diabetes clinic	*n* = 90 Patients with type 1 diabetes (≥5 y duration), active diabetic nephropathy	Int: *n* = 45 (25/20) Age: 12.4 ± 3.4 yCon: *n* = 45 (22/23) Age: 13.3 ± 2.8 y	Int: carnosineCon: placebo1 g · d^−1^ for 12 wk (2 × 500-mg doses)	FG (mg · dL^−1^)HbA1c (%)
Houjeghani et al. (21), Iran	RCT, hospital	*n* = 54 Patients with type 2 diabetes, not receiving exogenous insulin	Int: *n* = 22 [13/10 (*sic*)]	Int: carnosine	FG (mg/dL)
			Age: 43 ± 7.6 y	Con: placebo (cellulose)	FI (*μ*IU · mL^−1^)
			BMI: 29.1 ± 5.3	1 g · d^−1^ for 12 wk (2 × 500-mg doses)	HbA1c (%)
			Con: *n* = 22 [9/12 (*sic*)]		HOMA-*β* (%)
			Age: 40.4 ± 5.1 y		HOMA-IR
			BMI: 28.3 ± 4.6		
Nealon et al. (50), Australia	RCT, university	*n* = 12 Patients with type 2 diabetes, not receiving exogenous insulin	Int: *n* = 7 (2/5)	Int: *β*-alanine	FG (mmol · L^−1^)
			Age: 62 ± 4.6 y	Con: placebo (maltodextrin)	FI (mU · L^−1^)
			BMI: 30.9 ± 2.5	4 g · d^−1^ for 4 wk (3 × 1334-mg doses)	HOMA-*β* (%)
			Con: *n* = 5 (1/4)		HOMA-IR
			Age: 66 ± 6.4 y		
			BMI: 35.2 ± 8.5		

1 Con, control group; FG, fasting glucose; FI, fasting insulin; HbA1c, glycated hemoglobin, HOMA-*β*, homeostatic model assessment for steady-state *β*-cell function; HOMA-IR, homeostatic model assessment for insulin resistance; Int, intervention group; RCT, randomized controlled trial.

**TABLE 3:** Characteristics and outcomes for included animal studies^1^

Study (reference), country	Population	Disease model and method	Intervention	Included outcomes
Albrecht et al. (18), Germany	Male BTBR *ob*/*ob* mice	Genetic modification to develop obesity, hyperglycemia, and insulin resistanceTreatment started at 6 wk old	Duration: 18 wk	FG (mg · dL^−1^)
	6 wk old		Int: carnosine, oral, dissolved in drinking water:	FI (ng/mL)
	*n* = 15 per group		*1*) 45 mg · kg bw^−1 .^ d^−1^	HbA1c (%)
			Con: no intervention	
Aldini et al. (52), Italy	Male Zucker obese *fa*/*fa* rats	Genetic modification to develop obesity, hyperglycemia, and insulin resistanceTreatment started at 6 wk old	Duration: 24 wk	FG (mmol · L^−1^)
	5 wk old		Int: l-carnosine, oral, dissolved in drinking water:	FI (pmol · L^−1^)
	*n* = 6 per group		*1*) 30 mg · kg bw^−1 .^ d^−1^	HOMA-IR
			Con: no intervention	
Al-Sawalha et al. (54), Jordan	Male Wistar rats	Dietary intervention to develop metabolic syndromeHFHC diet (sucrose, margarine, etc.) and 20% sucrose added to drinking waterTreatment started alongside diet	Duration: 16 wk	FG (mg · dL^−1^)
	Young adults		Int: carnosine, injection:	FI (pg/mL)
	*n* = 10 per group		*1*) 250 mg · kg bw^−1^ × 5 per week	
			Con: vehicle only, injection	
Aydin et al. (57), Turkey	Male Wistar rats	Single STZ injection: 40 mg · kg bw^−1^	Duration: 4 wk	FG (mg · dL^−1^)
	3–4 mo old*n* = 8 per group	HF diet for 12 wk: 4 wk prior to and 8 wk following STZ injection	Int: carnosine, injection: *1*) 250 mg · kg bw^−1^ × 5 per weekCon: vehicle only, injection	HbA1c (%)
			Glucose >200 mg · dL^−1^ (11.1 mmol ·L^−1^) considered diabeticTreatment started 4 wk after STZ injection	
Barca et al. (58), Italy	Male C57BL/6JB6 mice	Single STZ injection: 200 mg · kg bw^−1^	Duration: 2 wk	FG (mg · dL^−1^)
	12 wk old*n* = 5 per group	Glucose ≥250 mg · dL^−1^ (13.9 mmol ·L^−1^) considered diabetic	Int: carnosine, oral, dissolved in drinking water: *1*) 1 g · L^−1^ (0.1% conc.)	
		Treatment started after disease induction	Con: no intervention	
Giriş et al. (55), Turkey	Male Sprague-Dawley ratsAge NR	Dietary intervention to develop hyperglycemia and insulin resistance	Duration: 8 wkInt: carnosine, oral, dissolved in drinking water:	FG (mg · dL^−1^) FI (μU/mL)
	*n* = 8 per group	High-fructose diet (60% fructose); isocaloric to the control group diet	*1*) 120—150 mg · kg bw^−1 .^ d^−1^Con: no intervention	HbA1c (%)HOMA-IR
		Treatment started alongside diet		
Hue et al. (53), Korea	Male C57BL/6J *db/db* mice7 wk old	Genetic modification to develop obesity, hyperglycemia, and insulin resistance	Duration: 8 wkInt: carnosine, oral administration:	FI (ng/mL)HbA1c (%)
	*n* = 10 per group	Glucose >350 mg · dL−1 (19.4 mmol ·L−1) considered diabetic	*1*) 6 mg · kg bw^−1 .^ d^−1^	
			*2*) 30 mg · kg bw^−1 .^ d^−1^	
		Treatment began after disease induction	*3*) 150 mg · kg bw^−1 .^ d^−1^	
			Con: saline	
Hue et al. (59), Korea	Male ICR (CD-1) mice	Single STZ injection: 120 mg · kg bw^−1^	Duration: 12 wk	HbA1c (%)
	5 wk old	Glucose >300 mg · dL^−1^ (16.7 mmol ·L^−1^) considered diabetic	Int: carnosine, oral administration:	
	*n* = 10 per group		*1*) 6 mg · kg bw^−1 .^ d^−1^	
		Treatment started after disease induction	*2*) 30 mg · kg bw^−1 .^ d^−1^	
			*3*) 150 mg · kg bw^−1 .^ d^−1^	
			Con: saline	
Liu et al. (60), China	Male C57BL/6J mice68 wk old	Multiple STZ injections: 50 mg · kg bw^−1^ for 5 consecutive days	Duration: 16 wk Int: carnosine, oral, dissolved in drinking water:	FG (mmol · L^−1^)
	*n* = 6 per group	Glucose >300 mg · dL^−1^ (16.7 mmol ·L^−1^) considered diabetic	* 1*) 1000 mg · kg bw^−1 .^ d^−1^Con: no intervention	
		Treatment started after disease induction		
Peters et al. (61), Germany	Male Sprague-Dawley rats	Single/double STZ injection: 50 mg · kg bw^−1^	Duration: 24 wk	HbA1c (%)
	Age NR *n* = 13 per group	Glucose >400 mg · dL^−1^ (22.2 mmol ·L^−1^) considered diabetic	Int: carnosine, oral, dissolved in drinking water: *1*) 1000 mg · kg bw^−1 .^ d^−1^	
		Unilateral nephrectomy performed 4 wk after STZ injection; treatment started postsurgery	Con: no intervention	
Pfister et al. (62), Germany	Male Wistar rats	Single STZ injection: 45 mg · kg bw^−1^	Duration: 26 wk	HbA1c (%)
	Age NR *n* = 7–8 per group	Glucose >250 mg · dL^−1^ (13.9 mmol ·L^−1^) considered diabetic	Int: carnosine, oral, dissolved in drinking water: *1*) 1000 mg · kg bw^−1 .^ d^−1^	
		Treatment started 1 wk after disease induction	Con: no intervention	
Riedl et al. (63), Germany	Male Wistar rats	Single STZ injection: 45 mg · kg bw^−1^	Duration: 12 wk	HbA1c (%)
	Age NR *n* = 7–9 per group	Glucose >250 mg · dL^−1^ (13.9 mmol ·L^−1^) considered diabetic	Int: carnosine, oral, dissolved in drinking water 1) 1000 mg · kg bw−1 . d−1	
		Treatment started 1 wk after disease induction	Con: no intervention	
Sauerhöfer et al. (19), Germany	Male and female	Genetic modification to develop obesity, hyperglycemia, and insulin resistance	Duration: 18 wk	FG (mg · dL^−1^)
	C57BL/6J Lepr*^db^ db*/*db* mice		Int: carnosine, oral, dissolved in drinking water:	FI (no units)
	4 wk old	Treatment started at 4 wk old	*1*) 0.9 g · L^−1^ (4 mmol · L^−1^)	HbA1c (%)
	*n* = 8–16 per group		Con: no intervention	
Soliman et al. (64), Egypt	Male albino rats	Single STZ injection: 40 mg · kg bw^−1^	Duration: 4 wk	FG (mg · dL^−1^)
	Age NR	Glucose >200 mg · dL^−1^ (11.1 mmol ·L^−1^) considered diabetic	Int: carnosine, injection, administered daily:	
	*n* = 10 per group		*1*) 100 mg · kg bw^−1 .^ d^−1^	
		Treatment started after disease induction	*2*) 200 mg · kg bw^−1 .^ d^−1^	
			Con: vehicle only, injection	
Stegen et al. (56), Belgium	Male Sprague-Dawley rats	Dietary intervention to develop obesity, hyperinsulinemia, and insulin resistance	Duration: 8 wk	FG (mmol · L^−1^)
	3 wk old		Int: carnosine, oral, dissolved in drinking water:	FI (pmol · L^−1^)
	*n* = 9 per group	Hypercaloric HF diet (60% fat)	*1*) 1697 mg · kg bw^−1 .^ d^−1^	HOMA-IR
		Treatment started alongside diet	Int: *β*-alanine, oral, dissolved in drinking water:	
			*2*) 933 mg · kg bw^−1 .^ d^−1^	
			Con: no intervention	
Yan et al. (65), Taiwan	Male BALB/cA mice3 wk old	Multiple STZ injections: 40 mg · kg bw^−1^ for five consecutive days	Duration: 6 wkInt: carnosine, oral, dissolved in drinking water:	FG (mmol · L^−1^)FI (nmol · L−1)
	*n* = 8 per group	Glucose >200 mg · dL^−1^ (11.1 mmol ·L^−1^) considered diabetic	*1*) 5 g · L^−1^ (0.5% conc.) Con: no intervention	
		Treatment started after disease induction		

1 bw, body weight; Con, control group; conc., concentration; FG, fasting glucose; FI, fasting insulin; HbA1c, glycated hemoglobin; HF, high-fat; HFHC, high-fat, high-carbohydrate; Int, intervention group; IR, insulin resistance; NR, not reported; STZ, streptozotocin.

Data from human studies included between-group pre- to postintervention changes, whereas rodent studies included between-group postintervention changes only (e.g., no baseline data were available), longitudinal repeated measures, and multiple treatment doses. Only single studies reported 2-h glucose following a GTT (20), homeostatic model assessment of insulin sensitivity (HOMA-*S*) (50), and C-peptide (18), so these outcomes were not included in the meta-analysis. We excluded 10 rodent studies that did not record glucose or insulin in the fasted state, or where the measurement type was unclear (66–75); some studies also recorded HbA1c, but this outcome was retained as it does not need to be recorded in the fasted state (62, 63). Two included studies were translated from Korean into English (53, 59). The supplementary information contains individual study data used in the analyses (**Supplemental Human** and **Animal Data**); and a list of all excluded full-text studies, including reasons for exclusion (Supplemental Methods).

### Risk of bias assessment

#### Study risk of bias

There were no differences in the risk of bias for individual outcomes within studies, so we allocated a single rating at the study level. Of the 4 human studies, 1 study showed some concerns with risk of bias (51), the remaining 3 studies showed high risk of bias (20, 21, 50) (**Supplemental Table 1**). This was due to a lack of information on adherence rates or no adjustment for nonadherence in analyses, which led to a high risk of bias for domain 2: bias due to deviations from the intended outcomes. No studies had a prespecified analyses plan, which led to some concerns for domain 5: bias in selection of the reported results. The risk of bias profile, however, was different across studies and 50% of items showed low risk of bias. Nearly all rodent studies had the same risk of bias profile: 60% unclear, 38% low, and 2% high risk of bias (**Supplemental Table 2**). The majority of criteria were scored as unclear due to reporting issues, which is consistent with risk of bias assessments in previous animal studies (37).

#### Outcome reporting bias

Three human studies reported prospective trial registration [(51), NCT02928250; (21), IRCT2016011211689N2; (50), ACTRN12613000273785], with a further human study not reporting trial registration in the manuscript [(20), NCT02011100]. All outcomes included in this review were explicitly preregistered in 2 studies (21, 50), whereas the remaining studies did not preregister HbA1c (51), fasting glucose (20, 51), fasting insulin, or HOMA-*β* outcomes (20). All studies showed internal consistency when comparing the methods with the reported results.

#### Small study and publication bias

Due to the number of available studies, we only assessed small study bias for fasting glucose and HbA1c in rodent studies. Visual inspection of funnel plots revealed no substantive asymmetries, such that we did not conduct a quantitative assessment (**Supplemental Figures 1** and **2**).

### Results of individual studies

Two human studies did not show any side effects from carnosine supplementation (20, 51), whereas the remaining studies did not report information on side effects (21, 50). Cumulative supplement intake in human studies ranged from 84 g to 168 g (carnosine) and 112 g (*β*-alanine), which translated to estimated relative cumulative intakes of 1.1 g · kg^–1^ to 1.7 g · kg^–1^ [carnosine (20, 21)] and 1.2 g · kg^–1^ [*β*-alanine (50)]. It was not possible to estimate the relative cumulative intake for Elbarbary et al. (51) as body weight was reported as a standard score. Relative cumulative intakes in rodent studies ranged from 315 mg · kg^–1^ to 182 g · kg^–1^ (carnosine) and 52.2 g · kg^–1^ (*β*-alanine). There were too few studies for each outcome to reliably explore the dose-response across human studies. Due to substantial variability, values from rodent studies were log-transformed prior to meta-regressions to assess the dose-response.

### Primary outcome: fasting glucose

#### Human studies

**FIGURE 2:**
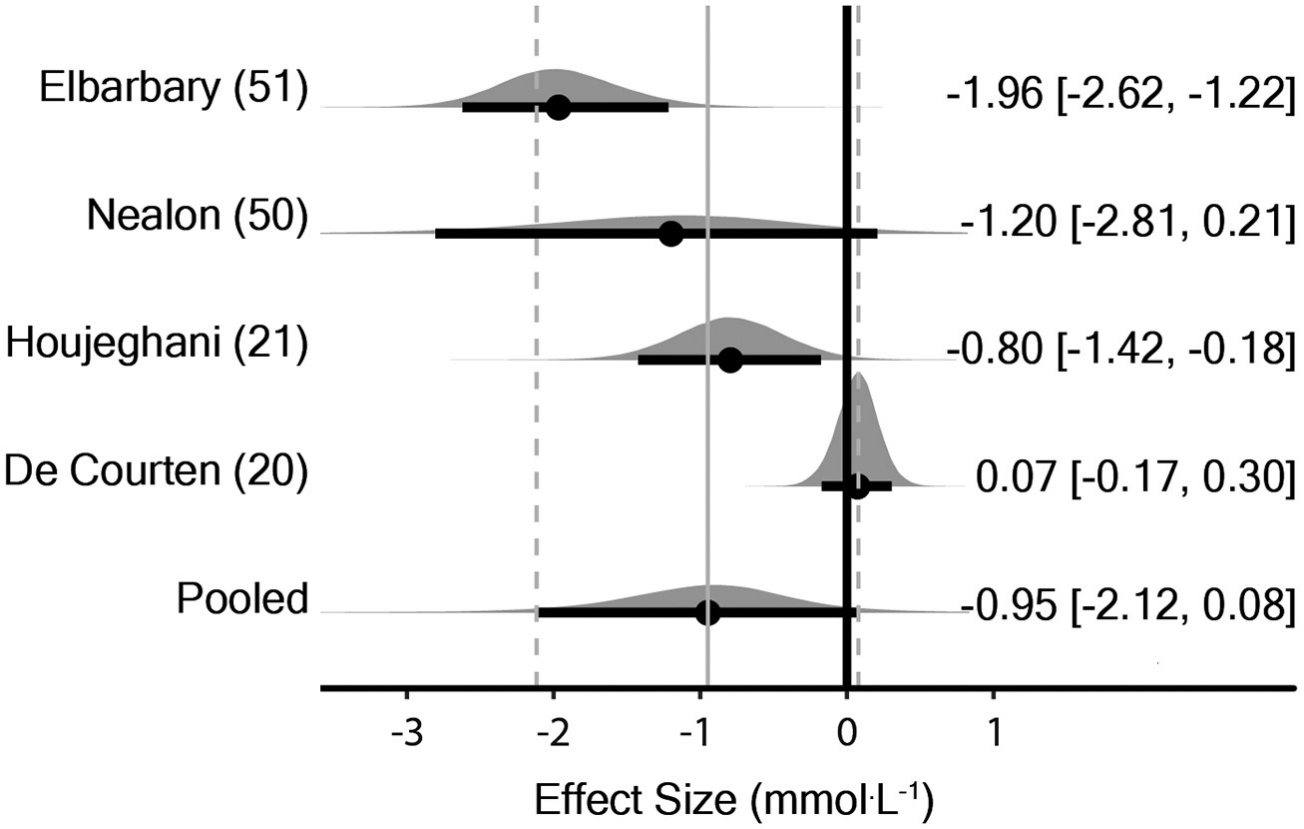
Bayesian forest plot of meta-analysis for fasting glucose in human studies. Each interval represents posterior “shrunken” estimates based on the random-effects model fitting and borrowing information across studies to reduce uncertainty. Circles represent the median value along with 90% credible intervals. Negative values show a reduction in fasting glucose in the intervention group compared with the control group. This analysis included 172 human participants (89 intervention/83 placebo).

The meta-analysis model (4 effect sizes from 4 studies) provided evidence for a decrease in fasting glucose with supplementation [MD_0.5_ = –0.95 mmol · L^–1^ (90% CrI: –2.12 to 0.08); τ_0.5_ = 0.97 mmol · L^–1^ (90% CrI: 0.48 to 2.3)] (**Figure 2**). A sensitivity analysis, removing data from studies where participants did not have elevated fasting glucose at baseline (20), was performed, which provided stronger evidence in favor of supplementation [MD_0.5_ = –1.5 mmol · L^–1^ (90% CrI: –2.49 to –0.54); τ_0.5_ = 0.54 mmol · L^–1^ (90% CrI: 0.05 to 1.99)]. The probability that the pooled effect size was less than or equal to the minimal important difference threshold (≥1 mmol · L^–1^ reduction) was estimated as *P* = 0.464 when including all studies and *P* = 0.841 when restricted to studies with elevated fasting glucose at baseline (where *P* closer to 1 indicates greater certainty based on posterior inferences).

#### Rodent studies

**FIGURE 3:**
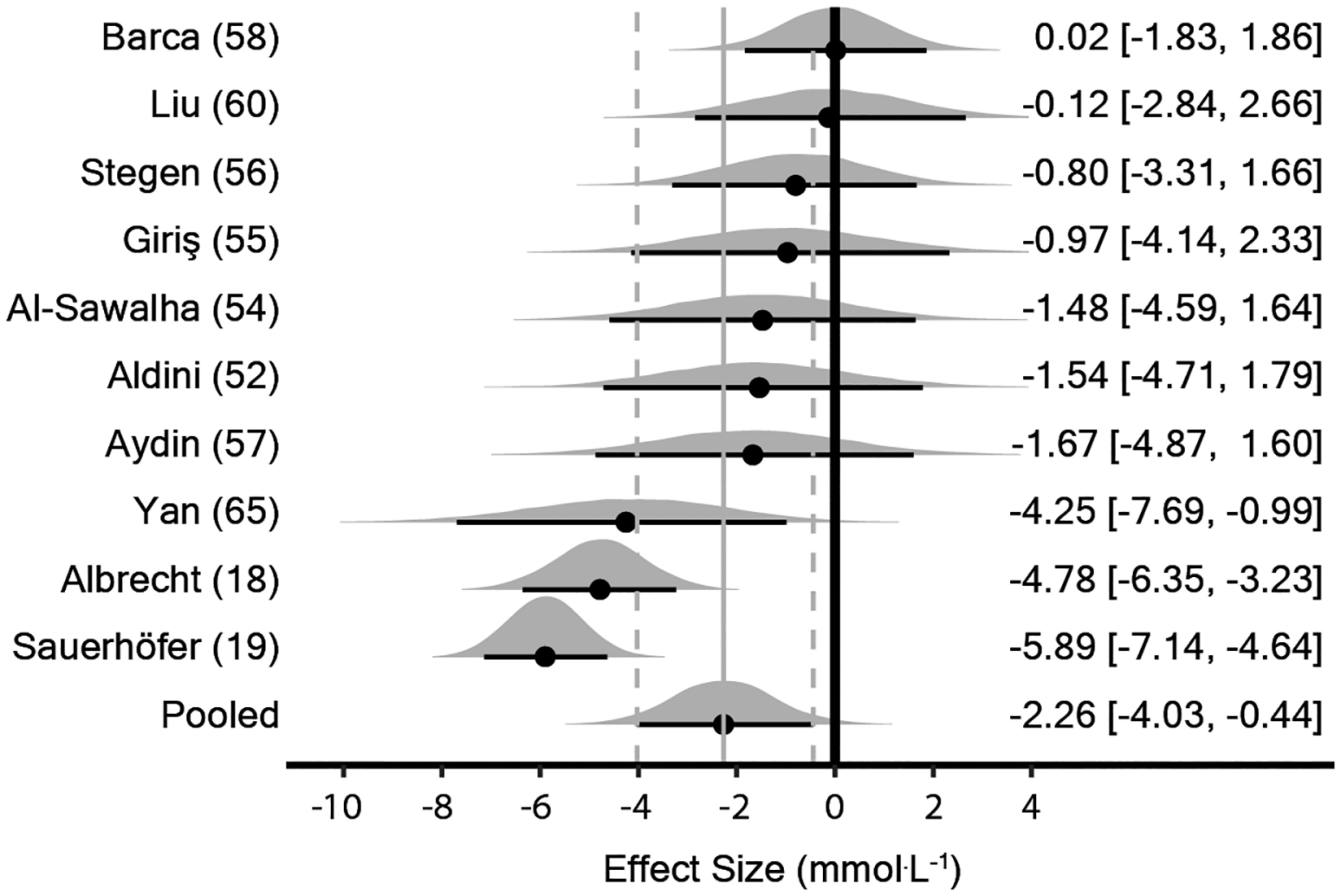
Bayesian forest plot of meta-analysis for fasting glucose in rodent studies. Each interval represents posterior “shrunken” estimates based on the random-effects model fitting and borrowing information across studies to reduce uncertainty. Circles represent the median value along with 90% credible intervals. Negative values show a reduction in fasting glucose in the intervention group compared with the control group. This analysis included 229 rodents (111 intervention/118 control).

Data from 1 study containing 2 large effect sizes [–20.6 and –20.5 (64)] were deemed outliers and excluded due to the effect sizes being several-fold higher than all other studies reporting this outcome. The meta-analysis model (45 effect sizes from 10 studies) provided evidence to support a decrease in fasting glucose with supplementation [MD_0.5_ = –2.26 mmol · L^–1^ (90% CrI: –4.03 to –0.44); τ_0.5_ = 2.7 mmol · L^–1^ (90% CrI: 1.6 to 4.7); intraclass correlation coefficient (ICC)_0.5_ = 0.33 (90% CrI: 0.16 to 0.53)] (**Figure 3**). A sensitivity analysis was performed removing data from studies where the method to induce disease did not elevate fasting glucose (56) and this provided support in favor of supplementation [MD_0.5_ = –2.58 mmol · L^–1^ (90% CrI: –4.50 to –0.61); τ_0.5_ = 2.81 mmol · L^–1^ (90% CrI: 1.6 to 5.0); ICC_0.5_ = 0.33 (90% CrI: 0.16 to 0.54)]. The dose-response analysis with meta-regression of effect size on the log-transformed cumulative dose showed greater decreases in fasting glucose with higher doses [*β*_0.5_ = –1.7 mmol · L^–1^ (90% CrI: –2.7 to –0.68); τ_0.5_ = 4.1 mmol · L^–1^ (90% CrI: 2.5 to 6.8)] (**Supplemental Figure 3**).

### Primary outcome: HbA1c

#### Human studies

**FIGURE 4:**
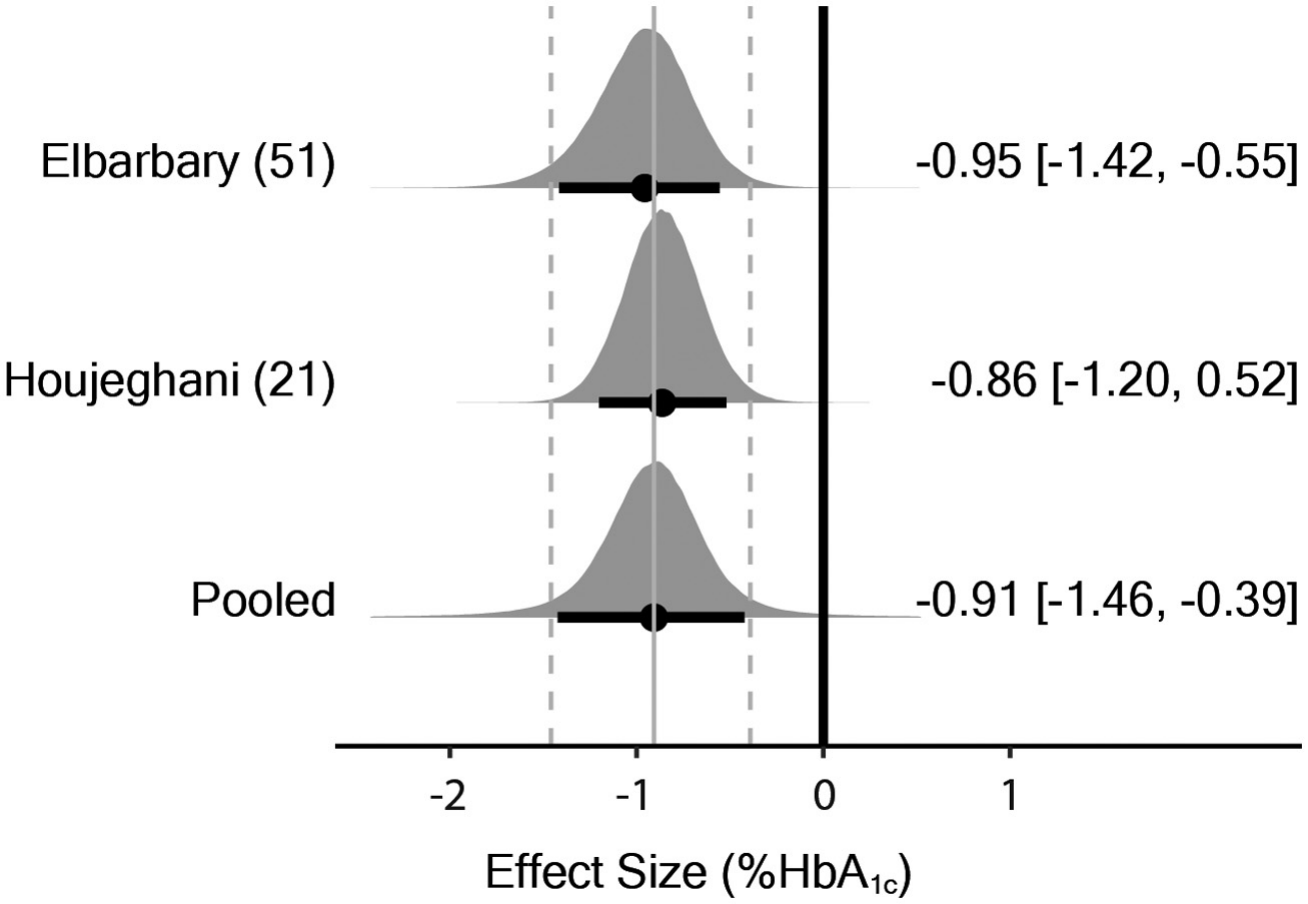
Bayesian forest plot of meta-analysis for HbA1c in human studies. Each interval represents posterior “shrunken” estimates based on the random-effects model fitting and borrowing information across studies to reduce uncertainty. Circles represent the median value along with 90% credible intervals. Negative values show a reduction in HbA1c in the intervention group compared with the control group. This analysis included 134 human participants (67 intervention/67 placebo). Both studies supplemented with carnosine. HbA1c (HbA_1c_), glycated hemoglobin.

The meta-analysis model (2 effect sizes from 2 studies) provided evidence for a decrease in HbA1c with supplementation [MD_0.5_ = –0.91% (90% CrI: –1.46 to –0.39); τ_0.5_ = 0.17% (90% CrI: 0.01 to 1.08)] (**Figure 4**). The probability that the pooled effect size was less than or equal to the minimal important difference threshold (≥0.5% reduction) was estimated as *P* = 0.921 (where *P* closer to 1 indicates greater certainty based on posterior inferences).

#### Rodent studies

**FIGURE 5:**
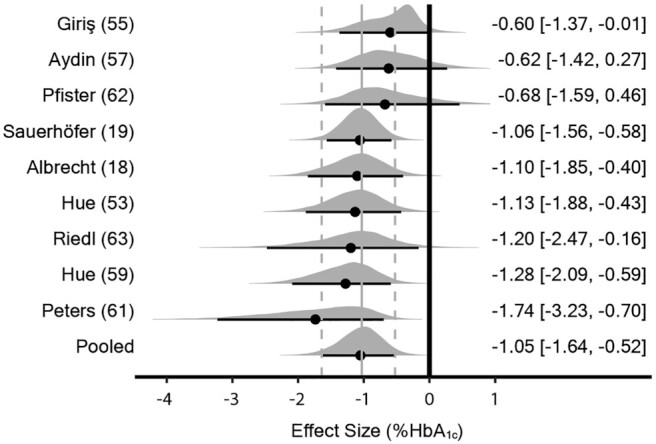
Bayesian forest plot of meta-analysis for HbA1c in rodent studies. Each interval represents posterior “shrunken” estimates based on the random-effects model fitting and borrowing information across studies to reduce uncertainty. Circles represent the median value along with 90% credible intervals. Negative values show a reduction in HbA1c in the intervention group compared with the control group. This analysis included 260 rodents (127 intervention/133 control). All studies supplemented with carnosine. HbA1c (HbA_1c_), glycated hemoglobin.

The meta-analysis model (16 effect sizes from 9 studies) provided evidence for a decrease in HbA1c with supplementation [random effects model: MD_0.5_ = –1.05% (90% CrI: –1.64 to –0.52); τ_0.5_ = 0.58% (90% CrI: 0.07 to 1.44); ICC_0.5_ = 0.11 (90% CrI: 0.00 to 0.53)] (**Figure 5**). Initially, no evidence of a dose response was obtained with meta-regression of effect size on the log-transformed cumulative dose [*β*_0.5_ = –0.03% (90% CrI: –0.32 to 0.27); τ_0.5_ = 0.62% (90% CrI: 0.08 to 1.52)] (**Supplemental Figure 4**). However, 1 effect size [cumulative dose: 182 g · kg · bw^–1^; effect size: 1.10 (62)] exhibited substantive leverage and removal of the point within a sensitivity analysis showed a possible dose-response effect in favor of greater decreases in HbA1c with higher doses [*β*_0.5_ = –0.18% (90% CrI: –0.50 to 0.13); τ_0.5_ = 0.57% (90% CrI: 0.08 to 1.39)] (Supplemental Figure 4).

### Additional outcomes: fasting insulin, HOMA-*β*, and HOMA-IR

#### Human studies

The meta-analysis models provided evidence of a small to medium effect for a decrease in HOMA-IR [SMD_0.5_ = –0.41 (90% CrI: –0.82 to –0.07); τ_0.5_ = 0.11 (90% CrI: 0.01 to 0.61); *P*(≤ –0.2) = 0.853; *P*(≤ –0.5) = 0.343; *P*(≤ –0.8) = 0.057] (**Supplemental Figure 5**); a small to medium effect in favor of decreased fasting insulin [SMD_0.5_ = –0.41 (90% CrI: –0.77 to –0.07); τ_0.5_ = 0.10 (90% CrI: 0.01 to 0.52); *P*(≤ –0.2) = 0.857; *P*(≤ –0.5) = 0.324; *P*(≤ –0.8) = 0.041] (**Supplemental Figure 6**); and a small effect in favor of decreased HOMA-*β* [SMD_0.5_ = –0.22 (90% CrI: –0.57 to 0.15); τ_0.5_ = 0.10 (90% CrI: 0.01 to 0.54); *P*(≤ –0.2) = 0.532; *P*(≤ –0.5) = 0.085; *P*(≤ –0.8) = 0.009] (**Supplemental Figure 7**) with supplementation.

#### Rodent studies

The meta-analysis models provided some evidence of a medium effect for decreased HOMA-IR [SMD_0.5_ = –0.63 (90% CrI: –1.98 to 0.65); τ_0.5_ = 0.72 (90% CrI: 0.06 to 2.84); ICC_0.5_ = 0.21 (90% CrI: 0.00 to 0.85); *P*(≤–0.2) = 0.745; *P*(≤–0.5) = 0.563; *P*(≤–0.8) = 0.364] (**Supplemental Figure 8**) and a small effect in favor of decreased fasting insulin [SMD_0.5_ = –0.31 (90% CrI: –1.33 to 0.57); τ_0.5_ = 1.03 (90% CrI: 0.16 to 2.72); ICC_0.5_ = 0.09 (90% CrI: 0.00 to 0.78); *P*(≤ –0.2) = 0.570; *P*(≤ –0.5) = 0.334; *P*(≤ –0.8) = 0.177] (**Supplemental Figure 9**) with supplementation.

### Sensitivity and subgroup analyses

Sensitivity analyses were performed to explore the robustness of the main analyses against outliers (rodent studies: fasting glucose and dose-response for HbA1c) and where the outcomes were not elevated at baseline (rodent studies: fasting glucose) or in the control group (rodent studies: fasting glucose). Data for these are presented in the previous sections. A sensitivity analysis for risk of bias was not performed due to no human studies being at low risk of bias and all rodent studies having similar risk of bias profiles. Only 1 rodent study supplemented with *β*-alanine, so meta-regressions for the effect of supplementation type were not performed. Instead, a sensitivity analysis was performed to show that the main results were robust to the removal of the *β*-alanine data from Stegen et al. (56): fasting glucose [MD_0.5_ = –2.36 mmol · L^–1^ (–4.16 to –0.49); τ_0.5_ = 2.73 mmol · L^–1^ (1.61 to 4.68); ICC = 0.34 (0.17 to 0.54)], HOMA-IR [SMD_0.5_ = –0.64 (90% CrI: –1.82 to 0.47); τ_0.5_ = 0.75 (90% CrI: 0.08 to 2.77); *P*(≤ –0.2) = 0.793; *P*(≤ –0.5) = 0.608; *P*(≤ –0.8) = 0.383], and fasting insulin [SMD_0.5_ = –0.33 (90% CrI: –1.50 to 0.57); τ_0.5_ = 1.07 (90% CrI: 0.15 to 2.80); ICC_0.5_ = 0.15 (90% CrI: 0.00 to 0.82); *P*(≤ –0.2) = 0.597; *P*(≤ –0.5) = 0.375; *P*(≤ –0.8) = 0.213]. Because there were no substantive differences, data from both supplementation groups in Stegen et al. (56) were combined into a single pooled effect size for the study.

### Certainty of evidence

There was moderate certainty in the effect estimates for human study outcomes. All outcomes were downgraded 1 level due to concerns with imprecision and risk of bias. It was decided not to rate down an additional level for the other outcomes, as there were no concerns with publication bias or inconsistency. There was very low certainty in the effect estimates for rodent study outcomes. All outcomes were downgraded 1 level, due to the prespecified criteria for indirectness and an additional level due to serious concerns with risk of bias and serious or very serious concerns with imprecision. The summary of findings table depicts the full GRADE assessment, with footnotes explaining each judgment (**Supplemental Table 3**).

## Discussion

### Summary of main findings

We included all available human and animal data to provide the most comprehensive assessment to date of the effects of carnosine and *β*-alanine supplementation on glycemic control and insulin resistance. Our main findings show that supplementation improves glycemic control across a range of disease types in humans (type 1 diabetic children, type 2 diabetic adults) and rodents (genetic models of obesity and diabetes, diet-induced metabolic syndrome, and pharmacological models of type 1 diabetes). As would be expected, there was no improvement in fasting glucose in normoglycemic populations. Of clinical relevance is the high probability that supplementation improves impaired fasting glucose (*P* = 0.841) and HbA1c (*P* = 0.921) beyond the minimal important difference thresholds (≥1 mmol · L^–1^ and ≥0.5% reduction). Data from animal studies support these findings, which strengthens our confidence in the effect. We also show evidence of a possible dose-response effect in animals, in favor of higher cumulative intakes causing greater reductions in fasting glucose and, possibly, HbA1c. The positive effects are driven primarily by carnosine supplementation, as only 1 human and 1 animal study supplemented with *β*-alanine (50, 56). There were insufficient data to assess the effect of supplementation on our other prespecified primary outcome: 2-h glucose following a GTT. Additional results show evidence of a small to medium effect in favor of supplementation reducing fasting insulin and HOMA-IR in humans. While there was some evidence in favor of supplementation reducing HOMA-*β* in humans, and fasting insulin and HOMA-IR in animals, it was not possible to rule out a neutral or negative effect for these outcomes.

### Proposed mechanisms

The inconsistent effects for fasting insulin across animal studies could be due to the relationship between hyperinsulinemia and insulin resistance, where an improvement in one may, paradoxically, lead to a decline in the other [for a commentary, see (76)]. Two long-term studies showed that supplementation attenuated the development of hyperglycemia in genetically modified mice (18, 19). Both studies reported an increase in fasting insulin, and 1 reported a 2-fold increase in C-peptide—a specific marker of insulin secretion (18). This suggests that carnosine might enhance insulin secretion from pancreatic *β*-cells, which may compensate for peripheral insulin resistance, leading to an improvement in glycemic control. Further, as supplementation began prior to disease development, it is possible that carnosine can play a role in preventing or delaying disease progression. One human study supports this: a subgroup of participants with impaired glucose tolerance displayed normal 2-h glucose and reduced 2-h insulin following supplementation (20). This suggests that carnosine might also improve postprandial glucose disposal, potentially by reducing peripheral insulin resistance. Consistent with these hypotheses, work from our research group showed that treatment with carnosine reverses glucolipotoxic inhibition of insulin secretion in isolated mouse islets and INS-1 pancreatic *β*-cells, as well as insulin-stimulated glucose uptake in C2C12 skeletal muscle cells (12). Together, this suggests that carnosine might exert beneficial effects in multiple tissues.

Improvements in glycemic control can protect organs and tissues from complications associated with diabetes. In type 1 diabetic children, Elbarbary et al. (51) showed a large decrease in plasma ɑ1-microglobulin (–44%) and the urinary albumin to creatinine ratio (UACR; –58%), which suggests that carnosine might have a protective effect on kidney function and may lower the risk of diabetic nephropathy. In support, several rodent studies showed reductions in the UACR (18, 52, 60, 61), as well as reductions in blood urea nitrogen and serum creatinine (60). Interestingly, these improvements occurred in 2 studies without a change in fasting glucose (52, 60). The studies showed an 11-fold and 4-fold increase in kidney carnosine concentrations following supplementation, supporting prior research that the human kidney has an intrinsic system for metabolizing carnosine (8). It is possible that some of the beneficial effects of carnosine occur directly in the kidney and may be independent of its actions on glycemic control—with positive outcomes in both type 1 and type 2 diabetes.

The most plausible mechanism by which carnosine provides a therapeutic effect is through its ability to form stable adducts with reactive carbonyl species, such as acrolein, 4-hydroxynoneal, and methylglyoxal (13, 77). These toxic products increase with diabetes severity and cause deleterious modifications to proteins, lipids, and DNA—inducing inflammation and insulin resistance, and impairing insulin secretion (12, 78–80). By scavenging these products, carnosine reduces their reactivity, allowing them to be safely metabolized or excreted from the body (14, 81, 82), which limits downstream formation of advanced glycation and advanced lipid-oxidation end products (AGEs and ALEs). Indeed, human and rodent studies showed supplementation protected against oxidative stress, lipid peroxidation, AGEs, and ALEs (18, 21, 51, 52, 55–57, 60, 62, 63, 65). It is also possible that carnosine works indirectly by activating the nuclear factor erythroid 2-related factor 2 (Nrf2) signaling cascade, which enhances endogenous antioxidant and anti-carbonylation defense systems [for a detailed review, see (83)].

There is a debate over the location of these actions. Some studies suggest that carnosine can act on reactive species and inflammatory markers in plasma, meaning that raising plasma carnosine would be key to any potential therapeutic effects (56). Although this might be true for rodents, where low carnosinase activity means that carnosine readily circulates in plasma and fasted values can increase 25-fold with supplementation (56), it seems unlikely to be as important in humans given that carnosinase is highly active in enterocytes and plasma, rapidly hydrolyzing carnosine (84, 85). As such, plasma carnosine concentrations remain below the limit of detection (11, 86). In a subgroup of individuals with low plasma carnosinase activity, a single dose of carnosine (60 mg · kg^–1^; 4.2 g for a 70-kg individual) increased plasma carnosine concentrations to a peak of 73.3 μM, before returning to baseline within 1–2 h (87).

Carnosine might instead act in human tissues that synthesize it in situ—those expressing carnosine synthase and transporters for *β*-alanine and histidine—and which play an important role in the pathogenesis of insulin resistance and diabetes. Recent genetic studies support the tissue–carnosine hypothesis: overexpression of cardio-specific ATP-grasp domain-containing protein 1 (ATPGD1; carnosine synthase) increased carnosine and anserine concentrations in the myocardium of mice, which reduced protein-aldehyde adducts and gave protection against ischemia reperfusion injury (88), whereas knockout of glutamic acid decarboxylase–like 1 (GADL1) reduced carnosine concentrations in the olfactory bulb and skeletal muscle, leading to increased markers of oxidative stress (89). Based on this evidence, increasing tissue carnosine stores should be the primary goal of supplementation. The included human studies, however, used a dose or duration that would cause only a modest increase in tissue carnosine stores (90, 91). de Courten et al. (20) was the only human study to quantify the change in tissue carnosine, reporting a 33% increase in skeletal muscle stores after supplementing with 2 g · d^–1^ carnosine for 12 wk. In contrast, nonclinical studies often supplement with 3.2 g · d^–1^ to 6.4 g · d^–1^ of *β*-alanine, whereby large cumulative intakes (24 wk; 1075.2 g total) can lead to a 2-fold increase in skeletal muscle carnosine content (92). It is worth noting that Nealon et al. (50) used a high *β*-alanine dose and showed improvements in glycemic control and insulin resistance in a 4-wk period. The comparably low relative cumulative intakes in human studies, coupled with the evidence of a dose-response in rodent studies, suggest that there could be room for further improvement in human outcomes. Future studies should consider the type, dose, and duration of supplementation.

While our study provides evidence in favor of supplementation, we recommend caution as the results require replication in large-sample, high-quality studies. The GRADE assessment showed our certainty in the effect estimates from human studies is moderate, which suggests the true effect is likely to be close to the estimate of the effect, but there is a possibility that it is substantially different (48). Further, the certainty in all outcomes from rodent studies was graded as very low, due to inconsistency, imprecision, and indirectness. Despite this, results from animal and human studies agree, which collectively adds weight to the main findings and shows that changes in rodent outcomes for glycemic control and insulin resistance may translate to humans. Given the inherent limitations in preclinical studies, however, caution is needed when interpreting the rodent outcomes for the purposes of choosing treatment options for humans.

### Limitations in the research

Our study highlights several limitations in the existing evidence base, which future studies can improve upon. No human studies were at a low risk of bias. To address this, researchers should provide clear information on randomization and allocation concealment, publish a prespecified statistical analysis plan within the trial registration, and rigorously assess and report adherence and blinding to the intervention. All human studies were of short duration (≤3 mo) with modest cumulative intakes; it is possible that longer-duration studies with higher cumulative intakes could lead to better clinical outcomes, consistent with the dose-response results in rodents. This is particularly important since diabetic complications develop over many years and may not be captured in short-duration studies. Several rodent studies did not contain the relevant information to assess the risk of bias; researchers should aim to satisfy the SYRCLE criteria and follow animal study reporting guidelines [e.g., the ARRIVE guidelines (93)]. Preclinical researchers should also design studies that translate to human clinical outcomes; several rodent studies were excluded from the current analysis because they did not record glucose or insulin in the fasted state. While certain disease models cannot be fasted for prolonged periods due to ethical concerns, it is often possible to conduct a short-term fast during the light phase in rodents that is equivalent to an overnight fast in humans (94). Our study focused upon surrogate outcomes involved in diabetes diagnosis and clinical decision making. These are important, although future RCTs should collect data on key patient-centered outcomes, such as disease progression rates, diabetic symptoms, hospital admissions, and diabetic complications.

### Conclusions

Our study provides evidence that supplementation with carnosine or *β*-alanine may reduce fasting glucose, HbA1c, and HOMA-IR in humans and rodents, and fasting insulin in humans. There is a need, however, for longer-term studies (>3 mo), in large samples, using dynamic methods to assess glycemic control and insulin resistance (e.g., GTTs and glucose clamp techniques). To improve the certainty in future findings, researchers should also explore dose-response effects in humans, whether treatment effects are the same for carnosine and *β*-alanine, and address shortcomings in study designs and reporting. Despite these caveats, our promising results indicate that carnosine and *β*-alanine supplementation remain viable therapeutics to improve glycemic control and insulin resistance in diabetes and its related conditions. The results of the current study provide a foundation upon which ongoing studies can be based, with the goal of defining whether this strategy is suitable for widespread population-level implementation.

## Supplementary Material

nmab087_Supplemental_Files

## Data Availability

Statistical code is available upon request. Data and other materials associated with the project are available in the Supplemental Methods, Supplemental Data, Supplemental Human Data, and Supplemental Animal Data files.

## Notes

The authors reported no funding received for this study. ED is supported by the Fundação de Amparo à Pesquisa do Estado de São Paulo (FAPESP; grant numbers: 2019/05616-6 and 2019/26899-6). GGA has been supported by FAPESP (grant numbers: 2014/11948-8 and 2019/25032-9) and by Coordenação de Aperfeiçoamento de Pessoal de Nível Superior (CAPES). MDT has received a British Council award to support a studentship focused on research into carnosine (grant number: 209524711).

Author disclosures: CS is the recipient of funding to support a PhD program of work from the Natural Alternatives International (NAI) and has received *β*-alanine supplements free of charge from NAI for use in experimental investigations; NAI have also supported open-access page charges for some manuscripts. GGA and ED have been co-investigators on projects partially supported by NAI. NAI have also supported open-access page charges for some manuscripts where GGA is the corresponding author. The other authors report no conflicts of interest.

Supplemental Figures 1–9, Supplemental Tables 1–3, Supplemental Methods, Supplemental Human Data, and Supplemental Animal Data are available from the link in the online posting of the article and from the same link in the online table of contents at https://academic.oup.com/advances/.

Abbreviations used: AGE, advanced glycation end product; ALE, advanced lipid-oxidation end product; bw, body weight; CrI, credible interval; GRADE, Grading of Recommendations Assessment, Development, and Evaluation; GTT, glucose-tolerance test; HbA1c, glycated hemoglobin; HCD, histidine-containing dipeptide; HOMA-β, homeostatic model assessment of β-cell function; HOMA-S, homeostatic model assessment of insulin sensitivity; ICC, intraclass correlation coefficient; MD, mean difference; PRESS, Peer Review Of Electronic Search Strategies; RCT, randomized controlled trial; SMD, standardized mean difference; SYRCLE, Systematic Review Center for Laboratory Animal Experimentation; UACR, urinary albumin to creatinine ratio.
