# Supplementary material for: Effect of Carnosine or β-Alanine Supplementation on Markers of Glycemic Control and Insulin Resistance in Humans and Animals: A Systematic Review and Meta-analysis
Source: Adv Nutr. 2021 Jul 31;12(6):2216–31. doi: 10.1093/advances/nmab087 (PMC8634390; doi:10.1093/advances/nmab087)
Supplement: nmab087_Supplemental_Files [file nmab087_supplemental_files.zip › 3.Supplementary_Data-270421_AdvNutr_Revision1.pdf]

Supplementary Data

Effect of carnosine or *β*-alanine supplementation on glycemic control and insulin resistance in humans and animals: a systematic review and meta-analysis

Joseph J Matthews, Eimear Dolan, Paul A Swinton, Livia Santos, Guilherme G Artioli, Mark D Turner, Kirsty J Elliott-Sale, Craig Sale

---

Contents

1. Risk of bias assessment in human studies
2. Risk of bias assessment in rodent studies
3. Funnel plots for assessment of small study bias
4. Dose response data for primary outcomes
5. Forest plots for additional outcomes
6. Certainty assessment: GRADE evidence profile and summary of findings table

Supplementary data: risk of bias assessment in human studies

Supplemental Table 1. Cochrane risk of bias 2.0 assessment of human studies.

Study	Intervention	Comparator	D1[1]	D2[2]	D3[3]	D4[4]	D5[5]	Overall
De Courten (20)	Carnosine	Placebo	!	-	-	+	!	-
Elbarbary (51)	Carnosine	Placebo	+	+	+	+	!	!
Houjeghani (21)	Carnosine	Placebo	+	-	!	+	!	-
Nealon (50)	β-alanine	Placebo	+	-	+	+	!	-

[1] *Domain 1: bias arising from the randomisation process*
[2] *Domain 2: bias due to deviations from the intended interventions*
[3] *Domain 3: bias due to missing outcome data*
[4] *Domain 4: bias in measurement of the outcome*
[5] *Domain 5: bias in selection of the reported results*
Key: green (+), *low risk of bias; yellow* (!), *some concerns; red* (-), *high risk of bias.*

Supplementary data: risk of bias assessment in rodent studies

Supplemental Table 2. SYRCLE risk of bias assessment of rodent studies.

Study	D1[1]	D2[2]	D3[3]	D4[4]	D5[5]	D6[6]	D7[7]	D8[8]	D9[9]	D10[10]
Albrecht (18)	!	+	!	!	!	!	!	+	+	+
Aldini (52)	!	+	!	!	!	!	!	+	+	+
Al-Sawalha (54)	!	+	!	!	!	!	!	+	+	+
Aydin (57)	!	+	!	!	!	!	!	+	+	+
Barca (58)	!	+	!	!	!	!	!	+	+	+
Giriş (55)	!	+	!	!	!	!	!	+	+	+
Hue (53)	!	+	!	!	!	!	!	+	+	+
Hue (59)	!	+	!	!	!	!	!	+	+	+
Liu (60)	!	+	!	!	!	!	!	+	+	+
Peters (61)	!	+	!	!	!	!	!	+	+	+
Pfister (62)	!	+	!	!	!	!	!	-	+	+
Riedl (63)	!	+	!	!	!	!	!	-	+	-
Sauerhöfer (19)	!	+	!	!	!	!	!	+	+	+
Soliman (64)	!	+	!	!	!	!	!	+	+	+
Stegen (56)	!	+	!	!	!	!	!	+	+	+
Yan (65)	!	+	!	!	!	!	!	+	+	+

[1]*Domain 1: random sequence generation (selection bias)*
[2]*Domain 2: baseline characteristics (selection bias)*
[3]*Domain 3: allocation concealment (selection bias)*
[4]*Domain 4: random housing (performance bias)*
[5]*Domain 5: blinding of trial caregivers (performance bias)*
[6]*Domain 6: random outcome assessment (detection bias)*
[7]*Domain 7: blinding of outcome assessment (detection bias)*
[8]*Domain 8: incomplete outcome data (attrition bias)*
[9]*Domain 9: selective reporting (reporting bias)*
[10]*Domain 10: Other bias*
Key: green (+), *low risk of bias; yellow* (!), *some concerns; red* (-), *high risk of bias.*

Supplementary data: funnel plots for assessment of small study bias

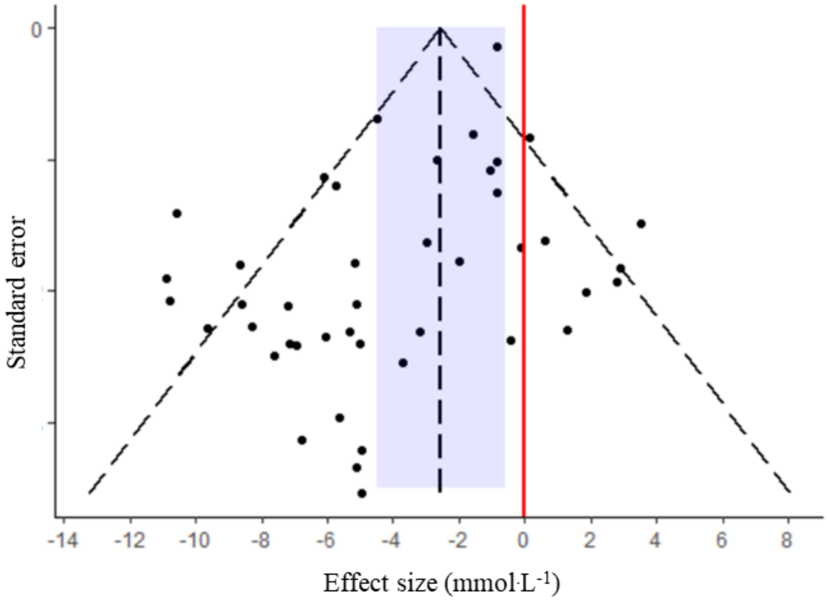

Supplemental Figure 1. Funnel plot of standard error vs effect size for fasting glucose in rodent studies.

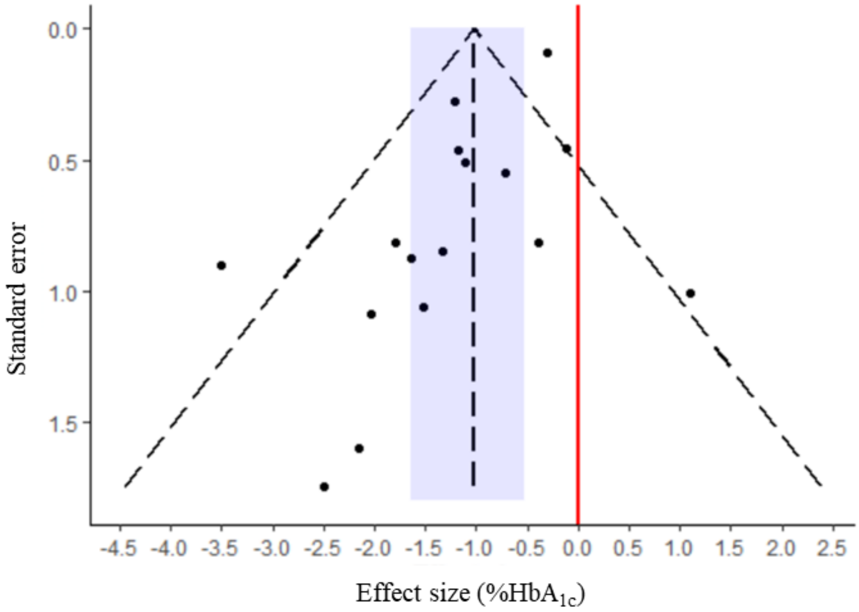

Supplemental Figure 2. Funnel plot of standard error vs effect size for HbA_{1c} in rodent studies.

Supplementary data: dose response data for primary outcomes

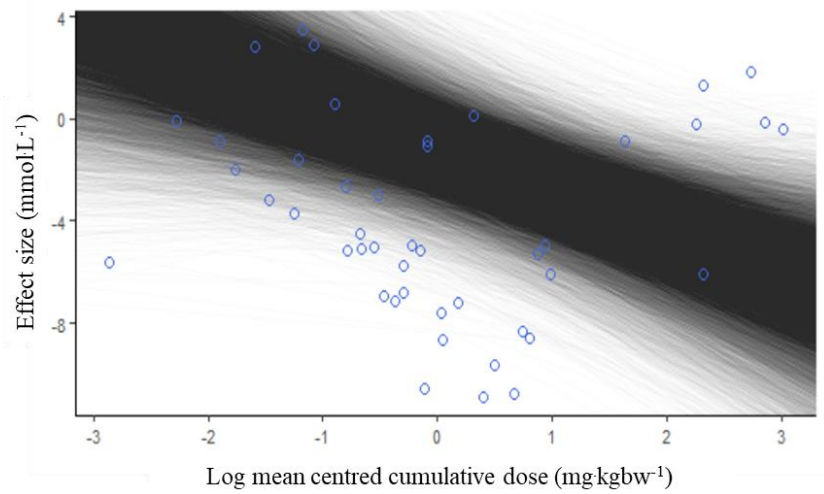

Supplemental Figure 3. Dose response data for fasting glucose in rodent studies following removal of one outlier (effect sizes: -20.6 and -20.5; (64)).

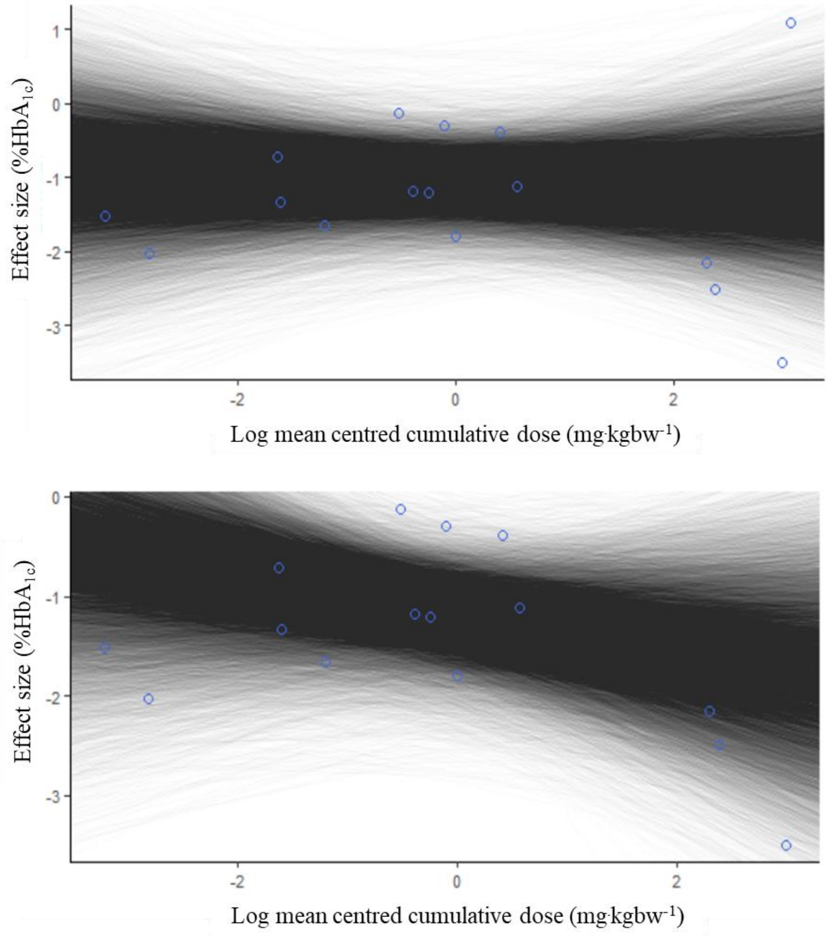

Supplemental Figure 4. Dose response data for HbA_{1c} in rodent studies before (top panel) and after (bottom panel) removal of one effect size from one study (effect size: 1.10; (62)).

Supplementary data: forest plots for additional outcomes

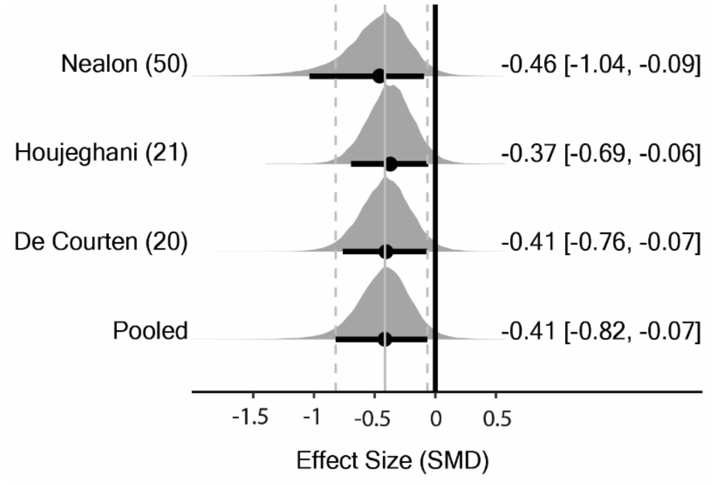

Supplemental Figure 5. Bayesian forest plot of meta-analysis for HOMA-IR in human studies. Each interval represents posterior “shrunken” estimates based on the random effects model fitting and borrowing information across studies to reduce uncertainty. Circles represent the median value along with 90% credible intervals. Negative values show a reduction in HOMA-IR in the intervention group compared to the control group: presented as standardised mean difference (effect sizes). This analysis included 82 human participants (44 intervention/38 placebo).

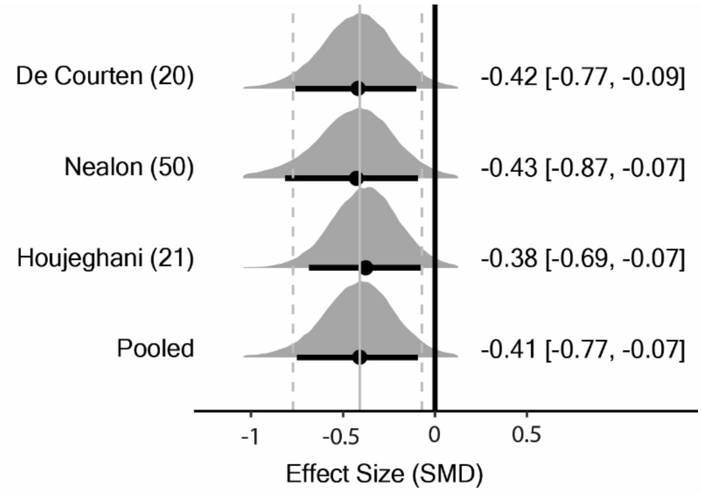

Supplemental Figure 6. Bayesian forest plot of meta-analysis for fasting insulin in human studies. Each interval represents posterior “shrunken” estimates based on the random effects model fitting and borrowing information across studies to reduce uncertainty. Circles represent the median value along with 90% credible intervals. Negative values show a reduction in fasting insulin in the intervention group compared to the control group: presented as standardised mean difference (effect sizes). This analysis included 82 human participants (44 intervention/38 placebo).

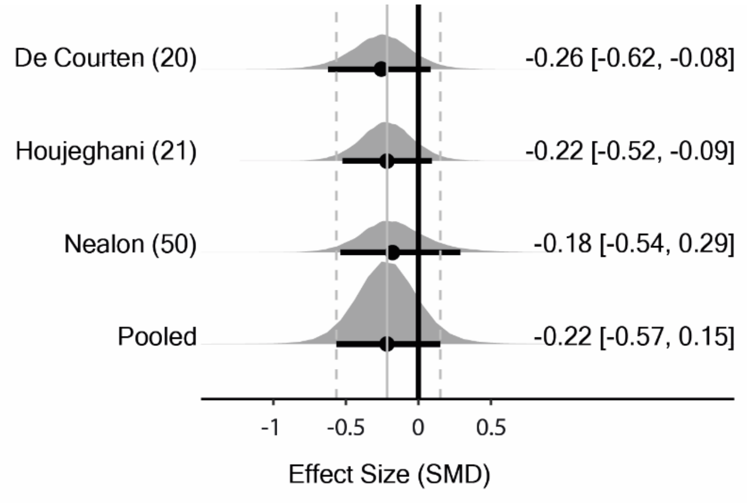

Supplemental Figure 7. Bayesian forest plot of meta-analysis for HOMA-β in human studies. Each interval represents posterior "shrunken" estimates based on the random effects model fitting and borrowing information across studies to reduce uncertainty. Circles represent the median value along with 90% credible intervals. Negative values show a reduction in HOMA-β in the intervention group compared to the control group: presented as standardised mean difference (effect sizes). This analysis included 82 human participants (44 intervention/38 placebo)

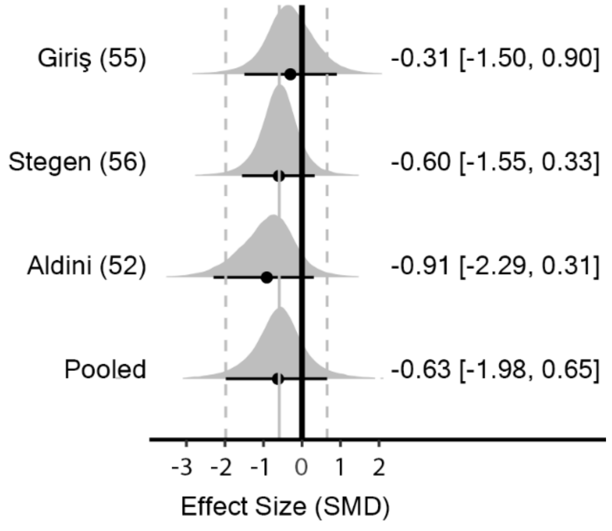

Supplemental Figure 8. Bayesian forest plot of meta-analysis for HOMA-IR in rodent studies. Each interval represents posterior "shrunken" estimates based on the random effects model fitting and borrowing information across studies to reduce uncertainty. Circles represent the median value along with 90% credible intervals. Negative values show a reduction in HOMA-IR in the intervention group compared to the control group: presented as standardised mean difference (effect sizes). This analysis included 64 rodents (32 intervention/32 control)

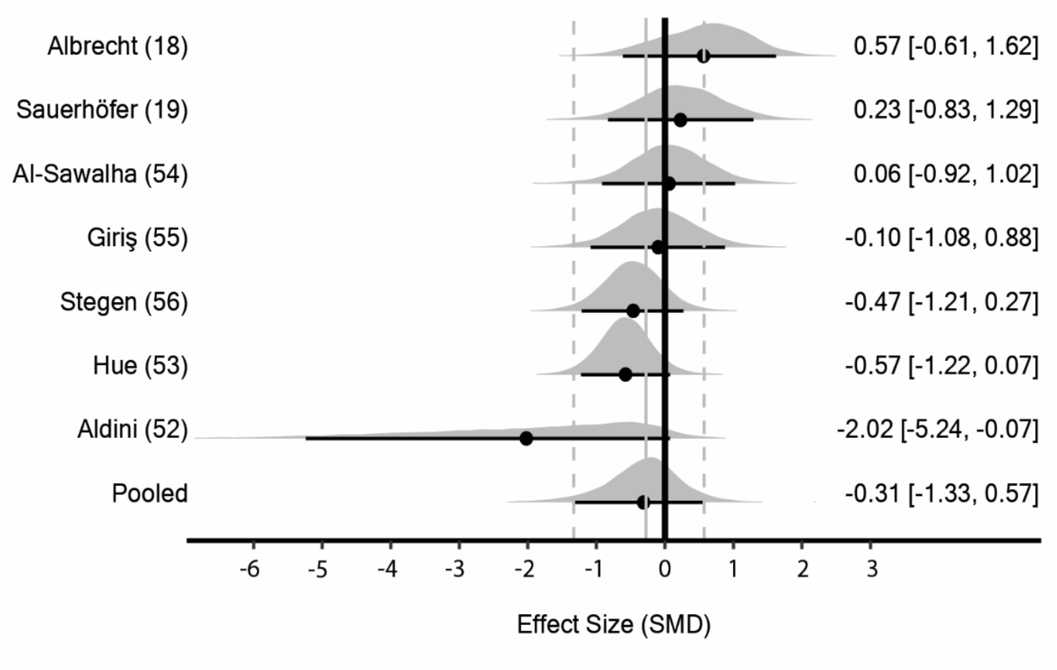

Supplemental Figure 9. Bayesian forest plot of meta-analysis for fasting insulin in rodent studies. Each interval represents posterior "shrunken" estimates based on the random effects model fitting and borrowing information across studies to reduce uncertainty. Circles represent the median value along with 90% credible intervals. Negative values show a reduction in fasting insulin in the intervention group compared to the control group: presented as standardised mean difference (effect sizes). This analysis included 203 rodents (102 intervention/101 control).

Supplementary data: certainty assessment

Supplemental Table 3. GRADE evidence profile and summary of findings table for human and rodent studies.

Certainty Assessment										
Sample	No. of Studies	Risk of Bias[1]	Inconsistency[2]	Indirectness[3]	Imprecision[4]	Publication Bias[5]	Number of Participants		Effect Size [90% CrI]	Certainty
							Intervention	Placebo		
Outcome: Fasting Glucose										
Human	4	Serious	Not Serious	Not Serious	Serious	Undetected	83	89	-0.95 $mmol \cdot L^{-1}$ [-2.1 to 0.08]	Moderate ⊕⊕⊕
Rodent	10	Serious	Serious	Very Serious	Serious	Undetected	118	111	-2.26 $mmol \cdot L^{-1}$ [-4.03 to -0.44]	Very Low ⊕
Outcome: HbA_{1c}										
Human	2	Serious	Not Serious	Not Serious	Serious	Undetected	67	67	-0.91% [-1.46 to -0.39]	Moderate ⊕⊕⊕
Rodent	9	Serious	Not Serious	Very Serious	Serious	Undetected	133	127	-1.05% [-1.64 to -0.52]	Very Low ⊕
Outcome: Fasting Insulin										
Human	3	Serious	Not Serious	Not Serious	Serious	Undetected	38	44	SMD -0.41 [-0.77 to -0.07]	Moderate ⊕⊕⊕
Rodent	7	Serious	Serious	Very Serious	Very Serious	Undetected	101	102	SMD -0.31 [-1.33 to 0.57]	Very Low ⊕
Outcome: HOMA-β										
Human	3	Serious	Not Serious	Not Serious	Serious	Undetected	38	44	SMD -0.22 [-0.57 to 0.15]	Moderate ⊕⊕⊕
Outcome: HOMA-IR										
Human	3	Serious	Not Serious	Not Serious	Serious	Undetected	38	44	SMD -0.41 [-0.82 to -0.07]	Moderate ⊕⊕⊕
Rodent	3	Serious	Not Serious	Very Serious	Very Serious	Undetected	32	32	SMD -0.63 [-1.98 to 0.65]	Very Low ⊕

Human studies

[1] *Graded serious due to no studies being at low risk of bias and several concerns with domain two and domain five.*

[2] *Graded not serious due to consistency with credible intervals; and the inconsistency in fasting glucose could be explained by baseline values across studies.*

[3] *Graded not serious due to narrow eligibility criteria, which selected for the population, intervention, and outcomes of interest.*

[4] *Graded serious due to the small, pooled sample size for each outcome.*

[5] *We could not detect this due to the small number of human studies.*

Rodent studies

[1] *Graded serious for all outcomes due to some concerns with risk of bias across studies.*

[2] *Graded serious for fasting glucose and fasting insulin due to large between study variability.*

[3] *Graded very serious for all outcomes due to the indirectness inherent in non-primate animal models.*

[4] *Graded serious for fasting glucose/HbA_{1c} due to the small, pooled sample size; very serious for fasting insulin/HOMA-IR due to the CrI including a large effect for harm and benefit.*

[5] *We did not detect evidence of publication bias for fasting glucose or HbA_{1c}, or for other outcomes due to the number of available studies.*
