# Supplementary material for: Effect of Carnosine or β-Alanine Supplementation on Markers of Glycemic Control and Insulin Resistance in Humans and Animals: A Systematic Review and Meta-analysis
Source: Adv Nutr. 2021 Jul 31;12(6):2216–31. doi: 10.1093/advances/nmab087 (PMC8634390; doi:10.1093/advances/nmab087)
Supplement: nmab087_Supplemental_Files [file nmab087_supplemental_files.zip › 4.Supplementary_Methods_270421_AdvNut_Revision1.pdf]

Online Supplementary Material

Supplementary Methods

Effect of carnosine or *β*-alanine supplementation on glycemic control and insulin resistance in humans and animals: a systematic review and meta-analysis

Joseph J Matthews, Eimear Dolan, Paul A Swinton, Livia Santos, Guilherme G Artioli, Mark D Turner, Kirsty J Elliott-Sale, Craig Sale

---

Contents

1. Full search strings for each database
2. Completed PRESS checklist
3. Excluded full text papers with reasons for exclusion

Supplemental methods 1: full search strings for each database

PubMed search strategy – https://pubmed.ncbi.nlm.nih.gov/

Search builder

1. “glucose metabolism disorders” [MeSH Terms]
2. “metabolic syndrome” [MeSH Terms]
3. “diabetes” [All Fields]
4. “prediabetes” [All Fields]
5. “hyperglycemia” [All Fields]
6. “hyperglycaemia” [All Fields]
7. “overweight” [MeSH Terms]
8. “overweight” [All Fields]
9. “obesity” [All Fields]
10. or/1-9
11. “carnosine” [Title/Abstract]
12. “beta-alanine” [Title/Abstract]
13. or/11-12
14. “insulin” [MeSH Terms]
15. “insulin” [All Fields]
16. “blood glucose” [MeSH Terms]
17. “fasting glucose” [All Fields]
18. “blood glucose” [All Fields]
19. “plasma glucose” [All Fields]
20. “glycemic” [All Fields]
21. “glycaemic” [All Fields]
22. “glycated hemoglobin A” [MeSH Terms]
23. “glycated haemoglobin A” [All Fields]
24. “HbA1c” [All Fields]
25. or/14-24
26. and/10, 13, 25

Filters or Limiters: None applied.

Date Conducted: December 31, 2020. Papers retrieved, n = 80.

**Overview**	
Full Search	((“glucose metabolism disorders” [MeSH Terms] OR “metabolic syndrome” [MeSH Terms] OR “diabetes” [All Fields] OR “prediabetes” [All Fields] OR “hyperglycemia” [All Fields] OR “hyperglycaemia” [All Fields] OR “overweight” [MeSH Terms] OR “overweight” [All Fields] OR “obesity” [All Fields]) AND ("carnosine" OR "beta-alanine"[Title/Abstract])) AND (“insulin” [MeSH Terms] OR “insulin” [All Fields] OR “blood glucose” [MeSH Terms] OR “fasting glucose” [All Fields] OR “blood glucose” [All Fields] OR “plasma glucose” [All Fields] OR “glycemic” [All Fields] OR “glycaemic” [All Fields] OR “glycated hemoglobin A” [MeSH Terms] OR “glycated haemoglobin A” [All Fields] OR “HbA1c” [All Fields])
**Breakdown**	
Population	“glucose metabolism disorders” [MeSH Terms] OR “metabolic syndrome” [MeSH Terms] OR “diabetes” [All Fields] OR “prediabetes” [All Fields] OR “hyperglycemia” [All Fields] OR “hyperglycaemia” [All Fields] OR “overweight” [MeSH Terms] OR “overweight” [All Fields] OR “obesity” [All Fields]
Intervention	"carnosine" OR "beta-alanine"[Title/Abstract]
Outcome	“insulin” [MeSH Terms] OR “insulin” [All Fields] OR “blood glucose” [MeSH Terms] OR “fasting glucose” [All Fields] OR “blood glucose” [All Fields] OR “plasma glucose” [All Fields] OR “glycemic” [All Fields] OR “glycaemic” [All Fields] OR “glycated hemoglobin A” [MeSH Terms] OR “glycated haemoglobin A” [All Fields] OR “HbA1c” [All Fields]

Scopus search strategy – https://www.scopus.com/

Search builder

1. “Metabolic syndrome” [ALL]
2. “Diabetes” [ALL]
3. “Prediabetes” [ALL]
4. “Hyperglycemia” [ALL]
5. “Hyperglycaemia” [ALL]
6. “Overweight” [ALL]
7. “Obesity” [ALL]
8. or/1-7
9. “Carnosine” [TITLE-ABS-KEY]
10. “Beta-alanine” [TITLE-ABS-KEY]
11. or/9-10
12. “Insulin” [ALL]
13. “Fasting glucose” [ALL]
14. “Blood glucose” [ALL]
15. “Plasma glucose” [ALL]
16. “Glycemic” [ALL]
17. “Glycaemic” [ALL]
18. “Glycated hemoglobin A” [ALL]
19. “Glycated haemoglobin A” [ALL]
20. “HbA1c” [ALL]
21. or/12-20
22. and/8, 11, 21

TITLE-ABS-KEY = Title, Abstract and Keywords, ALL = All Fields.
Filters or Limiters: Document type “Article”.
Date Conducted: December 31, 2020. Papers retrieved, n = 452.

**Overview**	
Full Search	(ALL("diabetes" OR "prediabetes" OR "hyperglycemia" OR "hyperglycaemia" OR "metabolic syndrome" OR "overweight" OR "obesity") AND TITLE-ABS-KEY("carnosine" OR "beta-alanine")AND ALL("insulin" OR "fasting glucose" OR "blood glucose" OR "plasma glucose" OR "glycemic" OR "glycaemic" OR "glycated haemoglobin A" OR "glycated hemoglobin A" OR "HbA1c")) AND (LIMIT-TO (DOCTYPE,"ar"))
**Breakdown**	
Population	“diabetes” [ALL] OR “prediabetes” [ALL] OR “hyperglycemia” [ALL] OR “hyperglycaemia” [ALL] OR “metabolic syndrome” [ALL] OR “overweight” [ALL] OR “obesity” [ALL]
Intervention	"carnosine" [TITLE-ABS-KEY] OR "beta-alanine" [TITLE-ABS-KEY]
Outcome	“insulin” [ALL] OR “fasting glucose” [ALL] OR “blood glucose” [ALL] OR “plasma glucose” [ALL] OR “glycemic” [ALL] OR “glycaemic” [ALL] OR “glycated hemoglobin A” [ALL] OR “glycated haemoglobin A” [ALL] OR “HbA1c” [ALL]

Web of Science search strategy – https://www.webofknowledge.com/

Search builder

1. TS=“carnosine” OR “beta-alanine”
2. TS=“carnosine” OR “beta-alanine”
3. TS=“carnosine” OR “beta-alanine”
4. or/1-3
5. TS=“metabolic syndrome”
6. TS=“diabetes”
7. TS=“prediabetes”
8. TS=“hyperglycemia”
9. TS=“hyperglycaemia”
10. TS=“overweight”
11. TS=“obesity”
12. or/5-11
13. TS=“insulin”
14. TS=“fasting glucose”
15. TS=“blood glucose”
16. TS=“plasma glucose”
17. TS=“glycemic”
18. TS=“glycaemic”
19. TS=“glycated hemoglobin A”
20. TS=“glycated haemoglobin A”
21. TS=“HbA1c”
22. or/13-21
23. and/4, 12, 22

TI = Title, AB = Abstract, AK = Author Keywords, TS = Topic.
Filters or Limiters: None applied.
Date Conducted: December 31, 2020. Papers retrieved, n = 179

**Overview**	
Full Search	TS=(“carnosine” OR “beta-alanine”) *AND* TS=(“diabetes” OR “prediabetes” OR “hyperglycemia” OR “hyperglycaemia” OR “metabolic syndrome” OR “overweight” OR “obesity”) *AND* TS=(“insulin” OR “fasting glucose” OR “blood glucose” OR “plasma glucose” OR “glycemic” OR “glycaemic” OR “glycated haemoglobin A” OR “glycated hemoglobin A” OR “HbA1c”)
**Breakdown**	
Population	TS=(“diabetes” OR “prediabetes” OR “hyperglycemia” OR “hyperglycaemia” OR “metabolic syndrome” OR “overweight” OR “obesity”)
Intervention	TS=(“carnosine” OR “beta-alanine”)
Outcome	TS=(“insulin” OR “fasting glucose” OR “blood glucose” OR “plasma glucose” OR “glycemic” OR “glycaemic” OR “glycated hemoglobin A” OR “glycated haemoglobin A” OR “HbA1c”)

CENTRAL search strategy – https://www.cochranelibrary.com/central

Search builder

1. “metabolic syndrome” [All Text]
2. “diabetes” [All Text]
3. “prediabetes” [All Text]
4. “hyperglycemia” [All Text]
5. “hyperglycaemia” [All Text]
6. “overweight” [All Text]
7. “obesity” [All Text]
8. or/1-7
9. “carnosine” [ti,ab,kw]
10. “beta-alanine” [ti,ab,kw]
11. or/9-10
12. “insulin” [All Text]
13. “fasting glucose” [All Text]
14. “blood glucose” [All Text]
15. “plasma glucose” [All Text]
16. “glycemic” [All Text]
17. “glycaemic” [All Text]
18. “glycated hemoglobin A” [All Text]
19. “glycated haemoglobin A” [All Text]
20. “HbA1c” [All Text]
21. or/12-20
22. and/8, 11, 21

ti = Title, ab = Abstract, kw = Keywords.
Filters or Limiters: “Trials” only.
Date Conducted: December 31, 2020. Papers retrieved, n = 36.

**Overview**	
Full Search	(“diabetes” OR “prediabetes” OR “hyperglycemia” OR “hyperglycaemia” OR “metabolic syndrome” OR “overweight” OR “obesity”) AND ("carnosine" OR "beta-alanine"):ti,ab,kw AND (“insulin” OR “fasting glucose” OR “blood glucose” OR “plasma glucose” OR “glycemic” OR “glycaemic” OR “glycated hemoglobin A” OR “glycated haemoglobin A” OR “HbA1c”)
**Breakdown**	
Population	“diabetes” OR “prediabetes” OR “hyperglycemia” OR “hyperglycaemia” OR “metabolic syndrome” OR “overweight” OR “obesity”
Intervention	“carnosine” OR “beta-alanine”:ti,ab,kw
Outcome	“insulin” OR “fasting glucose” OR “blood glucose” OR “plasma glucose” OR “glycemic” OR “glycaemic” OR “glycated hemoglobin A” OR “glycated haemoglobin A” OR “HbA1c”

ProQuest search strategy – https://search.proquest.com/advanced

Search builder

1. “metabolic syndrome” [All Text]
2. “diabetes” [All Text]
3. “prediabetes” [All Text]
4. “hyperglycemia” [All Text]
5. “hyperglycaemia” [All Text]
6. “overweight” [All Text]
7. “obesity” [All Text]
8. or/1-7
9. “carnosine” [ab]
10. “beta-alanine” [ab]
11. or/9-10
12. “insulin” [All Text]
13. “fasting glucose” [All Text]
14. “blood glucose” [All Text]
15. “plasma glucose” [All Text]
16. “glycemic” [All Text]
17. “glycaemic” [All Text]
18. “glycated hemoglobin A” [All Text]
19. “glycated haemoglobin A” [All Text]
20. “HbA1c” [All Text]
21. or/12-20
22. and/8, 11, 21

ab = Abstract.
Filters or Limiters: None applied.
Date Conducted: December 31, 2020. Papers retrieved, n = 200.

**Overview**	
Full Search	("diabetes" OR "prediabetes" OR "hyperglycemia" OR "hyperglycaemia" OR "metabolic syndrome" OR "overweight" OR "obesity") AND ab ("carnosine" OR "beta-alanine") AND ("insulin" OR "fasting glucose" OR "blood glucose" OR "plasma glucose" OR "glycemic" OR "glycaemic" OR "glycated hemoglobin A" OR "glycated haemoglobin A" OR "HbA1c")
**Breakdown**	
Population	(“diabetes” OR “prediabetes” OR “hyperglycemia” OR “hyperglycaemia” OR “metabolic syndrome” OR “overweight” OR “obesity”)
Intervention	ab(“carnosine” OR “beta-alanine”)
Outcome	(“insulin” OR “fasting glucose” OR “blood glucose” OR “plasma glucose” OR “glycemic” OR “glycaemic” OR “glycated hemoglobin A” OR “glycated haemoglobin A” OR “HbA1c”)

CINAHL Complete search strategy – https://health.ebsco.com/products/cinahl-complete/allied-health-nursing

Search builder

1. “metabolic syndrome” [TX]
2. “diabetes” [TX]
3. “prediabetes” [TX]
4. “hyperglycemia” [TX]
5. “hyperglycaemia” [TX]
6. “overweight” [TX]
7. “obesity” [TX]
8. or/1-7
9. “carnosine” [TX]
10. “beta-alanine” [TX]
11. or/9-10
12. “insulin” [TX]
13. “fasting glucose” [TX]
14. “blood glucose” [TX]
15. “plasma glucose” [TX]
16. “glycemic” [TX]
17. “glycaemic” [TX]
18. “glycated hemoglobin A” [TX]
19. “glycated haemoglobin A” [TX]
20. “HbA1c” [TX]
21. or/12-20
22. and/8, 11, 21

AB = Abstract, TX = All Text.
Filters or Limiters: None applied.
Date Conducted: December 31, 2020. Papers retrieved, n = 19.

**Overview**	
Full Search	TX (“diabetes” OR “prediabetes” OR “hyperglycemia” OR “hyperglycaemia” OR “metabolic syndrome” OR “overweight” OR “obesity”) AND AB (“carnosine” OR “beta-alanine”) AND TX (“insulin” OR “fasting glucose” OR “blood glucose” OR “plasma glucose” OR “glycemic” OR “glycaemic” OR “glycated hemoglobin A” OR “glycated haemoglobin A” OR “HbA1c”)
**Breakdown**	
Population	TX (“diabetes” OR “prediabetes” OR “hyperglycemia” OR “hyperglycaemia” OR “metabolic syndrome” OR “overweight” OR “obesity”)
Intervention	AB (“carnosine” OR “beta-alanine”)
Outcome	TX (“insulin” OR “fasting glucose” OR “blood glucose” OR “plasma glucose” OR “glycemic” OR “glycaemic” OR “glycated hemoglobin A” OR “glycated haemoglobin A” OR “HbA1c”)

Supplemental methods 2: completed PRESS checklist

***PRESS Guideline* — search submission & peer review assessment**

Search submission: this section to be filled in by the searcher

Searcher: Joseph J Matthews **E-mail:** joseph.matthews2016@my.ntu.ac.uk

Date submitted: 17/06/2020 **Date requested by:** 24/06/2020

Systematic review title: The effect of carnosine or β-alanine supplementation on markers of glycaemic control and insulin resistance in human and animal studies: a protocol for a systematic review and meta-analysis

This search strategy is …

**X**	My PRIMARY (core) database strategy — First time submitting a strategy for search question and database
	My PRIMARY (core) strategy — Follow-up review NOT the first time submitting a strategy for search question and database. If this is a response to peer review, itemize the changes made to the review suggestions
	SECONDARY search strategy— First time submitting a strategy for search question and database
	SECONDARY search strategy — NOT the first time submitting a strategy for search question and database. If this is a response to peer review, itemize the changes made to the review suggestions

Database

PubMed (https://pubmed.ncbi.nlm.nih.gov/)

Interface

PubMed (https://pubmed.ncbi.nlm.nih.gov/)

Research question

The purpose of this systematic review and meta-analysis is to evaluate the effect of carnosine or β-alanine supplementation on markers of glycaemic control and insulin resistance in humans and animals.

Overview of PICOS eligibility criteria.

Participants	Humans with type I diabetes, type II diabetes, prediabetes, gestational diabetes, impaired fasting glucose, or impaired glucose tolerance (according to WHO guidelines: WHO, 2006; 2011); or with overweight/obesity (BMI $\geq$25 kg·m^{2}) where the relevant outcomes were collected and reported.<br><br>Animal studies using a diabetes-related disease model (see human criteria above); or overweight/obese animals where the relevant outcomes were collected and reported.<br><br>No restriction on age or comorbidities; or on the methods used to induce disease in animal studies.
Intervention	Supplementation with carnosine or β-alanine. We excluded studies that used a multi-ingredient supplement intervention.<br><br>Human studies included oral administration only, whereas animal studies also included administration by other means (*e.g.*, intraperitoneal or intravenous injection).
Comparator	Comparisons for human studies were between placebo and the experimental intervention.<br><br>Comparisons for animal studies were between placebo or control (no intervention) and the experimental intervention.<br><br>We excluded studies without a control or placebo group.
Outcomes	Outcomes relating to glycaemic control and insulin resistance: fasting glucose, HbA_{1c}, 2-hour glucose following a GTT (see supplementary information for a full list).
Study Designs	Studies were limited to non-randomised and RCTs, including cluster RCTs. We excluded cohort studies, cross-sectional studies, case series, case reports, commentary, and review articles.

BMI, body mass index; GGT, glucose tolerance test; RCTs, randomised controlled trials; WHO, World Health Organization.

Was a search filter applied?

Yes No ■

Please copy and paste your search strategy here, exactly as run, including the number of hits per line. **Hits per line listed in the box below.**

Preliminary PubMed search strategy

Search builder

14. "glucose metabolism disorders" [MeSH Terms]
15. "metabolic syndrome" [MeSH Terms]
16. "diabetes" [All Fields]
17. "prediabetes" [All Fields]
18. "hyperglycemia" [All Fields]
19. "hyperglycaemia" [All Fields]
20. "overweight" [MeSH Terms]
21. "overweight" [All Fields]
22. "obesity" [All Fields]
23. or/1-9
24. "carnosine" [Title/Abstract]
25. "beta-alanine" [Title/Abstract]
26. or/11-12
27. "insulin" [MeSH Terms]
28. "insulin" [All Fields]
29. "blood glucose" [MeSH Terms]
30. "fasting glucose" [All Fields]
31. "blood glucose" [All Fields]
32. "plasma glucose" [All Fields]
33. "glycemic" [All Fields]
34. "glycaemic" [All Fields]
35. "glycated hemoglobin A" [MeSH Terms]
36. "glycated haemoglobin A" [All Fields]
37. "HbA1c" [All Fields]
38. or/14-24
39. and/10, 13, 25

Filters or Limiters: None applied.

Date Conducted: June 24, 2020.

**Overview**	
Full Search<br>(hits: n=73)	(("glucose metabolism disorders" [MeSH Terms] OR "metabolic syndrome" [MeSH Terms] OR "diabetes" [All Fields] OR "prediabetes" [All Fields] OR "hyperglycemia" [All Fields] OR "hyperglycaemia" [All Fields] OR "overweight" [MeSH Terms] OR "overweight" [All Fields] OR "obesity" [All Fields]) AND ("carnosine" OR "beta-alanine"[Title/Abstract])) AND ("insulin" [MeSH Terms] OR "insulin" [All Fields] OR "blood glucose" [MeSH Terms] OR "fasting glucose" [All Fields] OR "blood glucose" [All Fields] OR "plasma glucose" [All Fields] OR "glycemic" [All Fields] OR "glycaemic" [All Fields] OR "glycated hemoglobin A" [MeSH Terms] OR "glycated haemoglobin A" [All Fields] OR "HbA1c" [All Fields])
**Breakdown**	
Population<br>(hits: n=1,005,522)	"glucose metabolism disorders" [MeSH Terms] OR "metabolic syndrome" [MeSH Terms] OR "diabetes" [All Fields] OR "prediabetes" [All Fields] OR "hyperglycemia" [All Fields] OR "hyperglycaemia" [All Fields] OR "overweight" [MeSH Terms] OR "overweight" [All Fields] OR "obesity" [All Fields]
Intervention<br>(hits: n=5,518)	"carnosine" OR "beta-alanine"[Title/Abstract]
Outcome<br>(hits: n=549,941)	"insulin" [MeSH Terms] OR "insulin" [All Fields] OR "blood glucose" [MeSH Terms] OR "fasting glucose" [All Fields] OR "blood glucose" [All Fields] OR "plasma glucose" [All Fields] OR "glycemic" [All Fields] OR "glycaemic" [All Fields] OR "glycated hemoglobin A" [MeSH Terms] OR "glycated haemoglobin A" [All Fields] OR "HbA1c" [All Fields]

PEER REVIEW ASSESSMENT: THIS SECTION TO BE FILLED IN BY THE REVIEWER

Reviewer:<br>Victoria Boskett<br>Research Support<br>Librarian	Email:<br>victoria.boskett@ntu.ac.uk	Date completed:<br>24/06/2020

1. TRANSLATION

A ---No revisions	X
B --- Revision(s) suggested	☐
C --- Revision(s) required	☐

If "B" or "C," please provide an explanation or example:

2. BOOLEAN AND PROXIMITY OPERATORS

A ---No revisions	X
B --- Revision(s) suggested	☐
C --- Revision(s) required	☐

If "B" or "C," please provide an explanation or example:

3. SUBJECT HEADINGS

A ---No revisions	X
B --- Revision(s) suggested	☐
C --- Revision(s) required	☐

If "B" or "C," please provide an explanation or example:

4. TEXT WORD SEARCHING

A ---No revisions	X
B --- Revision(s)suggested	☐
C --- Revision(s) required	☐

If "B" or "C," please provide an explanation or example:

5. SPELLING, SYNTAX, AND LINE NUMBERS

A ---No revisions	X
B --- Revision(s)suggested	
C --- Revision(s) required	

If "B" or "C," please provide an explanation or example:

6. LIMITS AND FILTERS

A ---No revisions	X
B --- Revision(s) suggested	
C --- Revision(s) required	

If "B" or "C," please provide an explanation or example:

7. OVERALL EVALUATION (Note: If one or more "revision required" is noted above, the response below must be "revisions required".)

A ---No revisions	X
B --- Revision(s) suggested	
C --- Revision(s) required	

Additional comments:

Supplementary methods 3: excluded full text papers with reasons for exclusion

The exclusion code for each article is provided prior to the first author's name.

[1] Inappropriate population (*n*=4)

[2] Inappropriate intervention (*n*=4)

[3] Inappropriate comparator (*n*=1)

[4] Inappropriate outcome (*n*=15)

[5] Inappropriate study design (*n*=1)

[6] Multiple inappropriate criteria (*n*=4)

[7] Could not obtain necessary data (*n*=1)

[8] Duplicate data set to another included study (*n*=9)

[4] Ahshin-Majd S, Zamani S, Kiamari T, Kiasalari Z, Baluchnejadmojarad T, Roghani M. Carnosine ameliorates cognitive deficits in streptozotocin-induced diabetic rats: Possible involved mechanisms. Peptides. 2016;86:102-11.

[1] Anderson EJ, Vistoli G, Katunga LA, Funai K, Regazzoni L, Monroe TB, Gilardoni E, Cannizzaro L, Colzani M, De Maddis D, Rossoni G. A carnosine analog mitigates metabolic disorders of obesity by reducing carbonyl stress. The Journal of clinical investigation. 2018;128(12):5280-93.

[4] Ansurudeen I, Sunkari VG, Grünler J, Peters V, Schmitt CP, Catrina SB, Brismar K, Forsberg EA. Carnosine enhances diabetic wound healing in the db/db mouse model of type 2 diabetes. Amino acids. 2012;43(1):127-34.

[8] Aydın AF, Küçükgergin C, Bingül İ, Doğan-Ekici I, Doğru-Abbasoğlu S, Uysal M. Effect of carnosine on renal function, oxidation and glycation products in the kidneys of high-fat diet/streptozotocin-induced diabetic rats. Experimental and Clinical Endocrinology & Diabetes. 2017;125(05):282-9.

[8] Baye E, Ukropec J, de Courten MP, Kurdiova T, Krumpolec P, Fernández-Real JM, Aldini G, Ukropcova B, de Courten B. Carnosine supplementation reduces plasma soluble transferrin receptor in healthy overweight or obese individuals: a pilot randomised trial. Amino acids. 2019;51(1):73-81.

[8] Baye E, Ukropec J, De Courten MP, Mousa A, Kurdiova T, Johnson J, Wilson K, Plebanski M, Aldini G, Ukropcova B, De Courten B. Carnosine supplementation improves serum resistin concentrations in overweight or obese otherwise healthy adults: a pilot randomized trial. Nutrients. 2018;10(9):1258.

[8] Baye E, Ukropec J, De Courten MP, Vallova S, Krumpolec P, Kurdiova T, Aldini G, Ukropcova B, De Courten B. Effect of carnosine supplementation on the plasma lipidome in overweight and obese adults: a pilot randomised controlled trial. Scientific reports. 2017;7(1):1-7.

[7] Brown BE, Kim CH, Torpy FR, Bursill CA, McRobb LS, Heather AK, Davies MJ, Van Reyk DM. Supplementation with carnosine decreases plasma triglycerides and modulates atherosclerotic plaque composition in diabetic apo E−/− mice. Atherosclerosis. 2014;232(2):403-9.

[8] Chang KJ. Effect of taurine and β-alanine on morphological changes of pancreas in streptozotocin-induced rats. In: Della Corte L, Huxtable RJ, Sgaragli G, Tipton KF (Eds.). *Taurine 4: Taurine and Excitable Tissues* (vol. 483). 2002 (pp. 571-577). Springer, Boston, MA.

[1] Chang KJ, Kwon W. Immunohistochemical localization of insulin in pancreatic β-Cells of taurine-supplemented or taurine-depleted diabetic rats. In: Della Corte L, Huxtable RJ, Sgaragli G, Tipton KF (Eds.). Taurine 4: Taurine and Excitable Tissues (vol. 483). 2002 (pp. 579-587). Springer, Boston, MA.

[4] Forsberg EA, Botusan IR, Wang J, Peters V, Ansurudeen I, Brismar K, Catrina SB. Carnosine decreases IGFBP1 production in db/db mice through suppression of HIF-1. J Endocrinol. 2015;225(3):159-67.

[4] Guo Y, Guo C, Ha W, Ding Z. Carnosine improves diabetic retinopathy via the MAPK/ERK pathway. Experimental and therapeutic medicine. 2019;17(4):2641-7.

[6] Han CH, Lin YS, Lee TL, Liang HJ, Hou WC. Asn-Trp dipeptides improve the oxidative stress and learning dysfunctions in D-galactose-induced BALB/c mice. Food & function. 2014;5(9):2228-36.

[8] Houjeghani S, Kheirouri S, Faraji E, Asghari Jafarabadi M, Jabbari M. Antioxidant Status, Lipid Peroxidation and Protein Oxidation in Type 2 Diabetic Patients; Beneficial Effects of Supplementation with Carnosine: A Randomized, Double-Blind, Placebo-Controlled Trial. Iranian Red Crescent Medical Journal. 2018;20(3).

[4] Korolkiewicz RP, Fujita A, Seto K, Suzuki K, Takeuchi K. Polaprezinc exerts a salutary effect on impaired healing of acute gastric lesions in diabetic rats. Digestive diseases and sciences. 2000;45(6):1200-9.

[4] Lee YT, Hsu CC, Lin MH, Liu KS, Yin MC. Histidine and carnosine delay diabetic deterioration in mice and protect human low density lipoprotein against oxidation and glycation. European journal of pharmacology. 2005;513(1-2):145-50.

[4] Marzook EA, Marzook FA, Abd El Moneim AE. Radioprotective and anti-diabetic effects of Costus speciosus and carnosine. Tropical journal of pharmaceutical research. 2020;19(1):121-7.

[2] Menini S, Iacobini C, Ricci C, Fantauzzi CB, Pugliese G. Protection from diabetes-induced atherosclerosis and renal disease by D-carnosine-octylester: effects of early vs late inhibition of advanced glycation end-products in Apoe-null mice. Diabetologia. 2015;58(4):845-53.

[8] Mesgari-Abbasi M, Valizadeh H, Mirzakhani N, Vahdatpour T. Protective effects of di-and tri-peptides containing proline, glycine, and leucine on liver enzymology and histopathology of diabetic mice. Archives of physiology and biochemistry. 2019;14:1-10.

[4] Mong MC, Chao CY, Yin MC. Histidine and carnosine alleviated hepatic steatosis in mice consumed high saturated fat diet. European journal of pharmacology. 2011;653(1-3):82-8.

[5] Nagai K, Niijima A, Yamano T, Otani H, Okumra N, Tsuruoka N, Nakai M, Kiso Y. Possible role of L-carnosine in the regulation of blood glucose through controlling autonomic nerves. Experimental biology and medicine. 2003;228(10):1138-45.

[1] Patel JP, Lau-Cam CA. Taurine attenuates pyridoxal-induced adrenomedullary catecholamine release and glycogenolysis in the rat. In: Oja SS, Saransaari P (Eds.). *Taurine 6*: *Taurine and Excitable Tissues* (vol. 583). 2006 (pp. 147-156). Springer, Boston, MA.

[4] Peters V, Schmitt CP, Zschocke J, Gross ML, Brismar K, Forsberg E. Carnosine treatment largely prevents alterations of renal carnosine metabolism in diabetic mice. Amino acids. 2012;42(6):2411-6.

[6] Qiong S, Hong Y, Jun-jie Z. Effects of carnosine and aspirin on thiol level in retina in diabetic rats. Chinese Journal of Experimental Ophthalmology. 2011;29(1):58-61.

[6] Qiu J, Hauske SJ, Zhang S, Rodriguez-Niño A, Albrecht T, Pastene DO, van den Born J, van Goor H, Ruf S, Kohlmann M, Teufel M. Identification and characterisation of carnostatine (SAN9812),

a potent and selective carnosinase (CN1) inhibitor with in vivo activity. Amino Acids. 2019;51(1):7-16.

[3] Rasul A, Latif N, Ali S, Ashfaq M, Mubarak S, Rehman I. Taurine supplementation normalizes skeletal muscle functions in type 2 diabetes rats. Pakistan Armed Forces Medical Journal. 2020;70(1):224-9.

[8] Regazzoni L, De Courten B, Garzon D, Altomare A, Marinello C, Jakubova M, Vallova S, Krumpolec P, Carini M, Ukropec J, Ukropcova B. A carnosine intervention study in overweight human volunteers: bioavailability and reactive carbonyl species sequestering effect. Scientific reports. 2016;6(1):1-3.

[4] Schaalan MF, Ramadan BK, Abd Elwahab AH. Synergistic effect of carnosine on browning of adipose tissue in exercised obese rats; a focus on circulating irisin levels. Journal of cellular physiology. 2018;233(6):5044-57.

[6] Shi Q, Yan H. Changes of the thiol levels in the corneas of the diabetic rats: effect of carnosine, aspirin and a combination eye drops. International journal of ophthalmology. 2010;3(3):211.

[2] Shi Q, Yan H, Li MY, Harding JJ. Effect of a combination of carnosine and aspirin eye drops on streptozotocin-induced diabetic cataract in rats. Molecular vision. 2009;15:2129.

[4] Vahdatpour T, Nokhodchi A, Zakeri-Milani P, Mesgari-Abbasi M, Ahmadi-Asl N, Valizadeh H. Leucine–glycine and carnosine dipeptides prevent diabetes induced by multiple low-doses of streptozotocin in an experimental model of adult mice. Journal of diabetes investigation. 2019;10(5):1177-88.

[8] Vahdatpour T, Valizadeh H, Mirzakhani N, Mesgari-Abbasi M. Renoprotective Effects of Di-and Tri-peptides Containing Proline, Glycine and Leucine in Diabetes Model of Adult Mice: Enzymology and Histopathology. International Journal of Peptide Research and Therapeutics. 2020;26(4):2345-54.

[2] Xu G, Gaul MD, Song F, Du F, Liang Y, DesJarlais RL, DiLoreto K, Shook B, Rentzeperis D, Santulli R, Eckardt A. Discovery of potent and orally bioavailable indazole-based glucagon receptor antagonists for the treatment of type 2 diabetes. Bioorganic & medicinal chemistry letters. 2019 Oct 15;29(20):126668.

[1] Yamano T, Niijima A, Iimori S, Tsuruoka N, Kiso Y, Nagai K. Effect of L-carnosine on the hyperglycemia caused by intracranial injection of 2-deoxy-D-glucose in rats. Neuroscience letters. 2001 Nov 2;313(1-2):78-82.

[2] Yan H, Guo Y, Zhang J, Ding Z, Ha W, Harding JJ. Effect of carnosine, aminoguanidine, and aspirin drops on the prevention of cataracts in diabetic rats. Molecular Vision. 2008;14:2282.

[4] Yang Y, Wang Y, Kong Y, Zhang X, Zhang H, Gang Y, Bai L. Carnosine prevents type 2 diabetes-induced osteoarthritis through the ROS/NF-κB pathway. Frontiers in pharmacology. 2018;9:598.

[4] Yapislar H, Aydogan S. Effect of carnosine on erythrocyte deformability in diabetic rats. Archives of physiology and biochemistry. 2012;118(5):265-72.

[4] Yay A, Akkuş D, Yapıslar H, Balcıoglu E, Sonmez MF, Ozdamar S. Antioxidant effect of carnosine treatment on renal oxidative stress in streptozotocin-induced diabetic rats. Biotechnic & Histochemistry. 2014;89(8):552-7.

[4] You JS, Lee YJ, Kim SY, Chang KJ, Cho HJ, Kim SH. N-(β-D-Xylopyranosyl) taurine sodium salt supplementation has beneficial effect on the hepatic antioxidant system of rats fed a high-fat diet and β-alanine. In: Marcinkiewicz J, Schaffer SW (Eds.). *Taurine 9* (vol. 803). 2015 (pp. 869-877). Springer, Boston, MA.
